# Super‐Resolution and High‐Data‐Density Acoustic Meta‐Hologram via Amplitude and Phase Coupling

**DOI:** 10.1002/advs.76251

**Published:** 2026-06-24

**Authors:** Xiao Guo, Xinzong Wang, Guoshen Tang, Haohan Zeng, Siqi Fan, Xinghao Hu, Youyu Mo, Zhenyu He, Tingting Li, Hui Xu, Jiao Shen, Haiyan Fan, Xiaoxiang Gao, Yifan Zhu, Hui Zhang, Badreddine Assouar

**Affiliations:** ^1^ Jiangsu Key Laboratory for Design and Manufacturing of Precision Medicine Equipment School of Mechanical Engineering Southeast University Nanjing China; ^2^ Key Laboratory of Underwater Acoustic Signal Processing (Southeast University) Ministry of Education Nanjing China; ^3^ State Key Laboratory of Digital Medical Engineering School of Biological Science and Medical Engineering Southeast University Nanjing China; ^4^ Institute of Microphysiological Systems Southeast University Nanjing China; ^5^ Institut Jean Lamour CNRS University of Lorraine Nancy France

**Keywords:** acoustic hologram, acoustic holographic imaging, acoustic metamaterials, acoustic metasurfaces, amplitude and phase coupling, high‐data‐density hologram, super‐resolution hologram

## Abstract

Image pixel density is a key factor limiting image clarity, while the scalar spatial bandwidth product (SBP) is the critical parameter determining the resolution of acoustic holographic imaging. Under finite SBP conditions, achieving high‐resolution imaging remains a significant challenge for current acoustic meta‐hologram techniques due to the constraints imposed by the diffraction limit of sound waves. To address the resolution and data density limitations of acoustic holography at the diffraction limit, we propose a super‐resolution and high data density holographic imaging method at low SBP based on amplitude‐phase coupling modulation mechanisms. By revealing the relationship between amplitude‐phase coupling and acoustic energy conservation, we derive a high‐resolution focused phase distribution suitable for low SBP under acoustic energy conservation constraints. Introducing coupled focused phases into the hologram enables super‐resolution acoustic field reconstruction and high‐data‐density information transmission at low SBP, providing a theoretical basis for overcoming the diffraction limit. We demonstrate the proposed concept of super‐resolution and high‐data‐density hologram through theoretical derivation, numerical simulation, and experimental validation. This super‐resolution holographic imaging method, based on amplitude‐phase coupling modulation under acoustic energy conservation constraints, provides a conceptually advanced technical approach for achieving high‐resolution acoustic hologram under low data density.

## Graphical abstract

A super‐resolution acoustic meta‐hologram utilizes amplitude‐phase coupling under energy conservation constraints to overcome the traditional diffraction limit. This conceptually advanced approach achieves high‐fidelity acoustic field reconstruction and dense information transmission, unlocking new potentials for high‐resolution acoustic imaging and communication under low spatial‐bandwidth products.

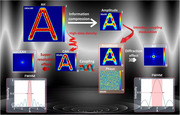

## 1 Introduction

Holographic technology [1] is a crucial technique for information storage [2, 3] and transmission [4], relying on spatial modulation and the reconstruction of wavefront amplitude and phase through interference and diffraction. By precisely recording and reproducing wavefront information, holographic technology provides an effective method for information transmission and flexible acoustic modulation. Information transmission data capacity is typically quantified by the scalar spatial bandwidth product (SBP) [1, 5]. Consequently, the scalar SBP of a hologram directly determines the spatial complexity of the acoustic field while being constrained by diffraction limits [6, 7]. Specifically, the information capacity of the acoustic field depends on the number of independently addressable pixels within the hologram [1]. A higher scalar SBP implies a larger acoustic field modulation area and finer acoustic field reconstruction capability. Therefore, achieving high‐resolution and high‐data‐density acoustic imaging under a fixed SBP holds significant research and application potential.

Holographic methods for wavefront modulation based on acoustic metamaterials [8, 9] and metasurfaces [10, 11] demonstrate tremendous potential. Through pure phase modulation [1] or combined amplitude‐phase modulation [12, 13], metasurfaces can achieve control over sound wave transmission [14, 15] and reflection [16], as well as focusing enhancement [17], vortex generation [18, 19, 20], and beam deflection [21]. These capabilities hold broad application prospects in acoustic tweezers [22], acoustic levitation [1, 23], acoustic traps [23], acoustic phase/amplitude imaging [24, 25], acoustic 3D printing [26], biomedical applications [27, 28], and information encryption [4, 29, 30, 31]. Traditional time‐reversal based acoustic holograms achieve good reconstruction of finite‐data target images under simultaneous amplitude and phase modulation. However, constrained by the diffraction limit, further enhancing image resolution and data density remains a significant challenge.

In recent years, to overcome the diffraction limit and further enhance image data density for improved imaging resolution, researchers have extensively explored utilizing evanescent waves [32] and acoustic meta‐lens [33, 34] for super‐resolution imaging and focusing. For instance, by exciting Fabry–Perot resonances within subwavelength structures, effective transmission and coupling of evanescent waves can be achieved, overcoming diffraction limitations in the near‐field range [32]. However, constrained by their physical mechanisms, the effective action distance of evanescent waves is typically limited to extremely short near‐field imaging distances. Given the significant limitations in practical applications, there is an urgent need to achieve far‐field super‐resolution imaging at the level of wavefront control mechanisms.

To address the aforementioned issues, this paper proposes a concept of coupling amplitude hologram (CAH) to realize super‐resolution and high‐data‐density acoustic meta‐hologram. Through theoretical derivation, we establish the relationship between amplitude‐phase coupling and acoustic energy conservation. Under energy conservation constraints, we employ the Horned Lizard Optimization Algorithm (HLOA) [35] to perform inverse inversion of the amplitude‐phase coupling process, thereby obtaining a coupled phase distribution capable of achieving superfocusing effects. Simultaneously taking advantage of the high‐precision amplitude‐phase independently modulated capability of holographic metasurfaces, this approach enables the reconstruction of complex acoustic fields beyond the diffraction limit. The CAH hologram proposed herein not only integrates the physical foundation of time‐reversal but also achieves breakthroughs at the diffraction limit at the wavelength scale through initial phase‐driven high‐amplitude phase shift control, significantly enhancing the resolution, information transmission density, and energy efficiency of reconstructed acoustic fields. Through systematic theoretical analysis, full‐wave numerical simulations, and experimental validation, we demonstrate the effectiveness and advanced nature of this method in achieving super‐resolution acoustic field focusing and high‐density information reconstruction. This work provides new insights for advancing acoustic holography toward applications requiring higher resolution and large information capacity.

## 2 Results

### 2.1 Super‐Resolution and High‐Data‐Density Acoustic Hologram of CAH

Holographic imaging can be achieved based on the principle of time‐reversal inversion. In this method, the target image is first discretized into an array of subwavelength‐scale pixels. Through the time‐reversal method, coherent superposition of wavefront components generated by each pixel on the image plane reconstructs the sound field distribution on the holographic plane. This sound field is described by the complex sound pressure $p_{j}=A_{j}\exp\left(i\phi_{j}\right)$, where *A_j_* and $\phi_{j}$ represent the amplitude and phase at that location, respectively. Specifically, the sound pressure at a point (*x_j_*,*y_j_*,*z_j_*) on the holographic plane can be expressed as

(1) $$p_{j}=\sum_{l=1}^{N}\frac{A_{0l}}{r_{l}}\exp\left[i\left(k_{0}r_{l}+\phi_{0l}\right)\right]\equiv A_{j}\exp\left(i\phi_{j}\right)$$

Here, N denotes the total number of pixels in the image. *A*_0*l*_ and $\phi_{0l}$ represent the amplitude and initial phase of the lth pixel at the image plane, respectively, while k0 is the wave number. *r_l_* denotes the Euclidean distance between the j‐th position on the holographic plane and the *l‐th* pixel at the image plane, given by the following equation

(2) $$r_{l}=\sqrt{{\left(x_{j}-x_{l}\right)}^{2}+{\left(y_{j}-y_{l}\right)}^{2}+{\left(z_{j}-z_{l}\right)}^{2}}\;$$

**FIGURE 1:**
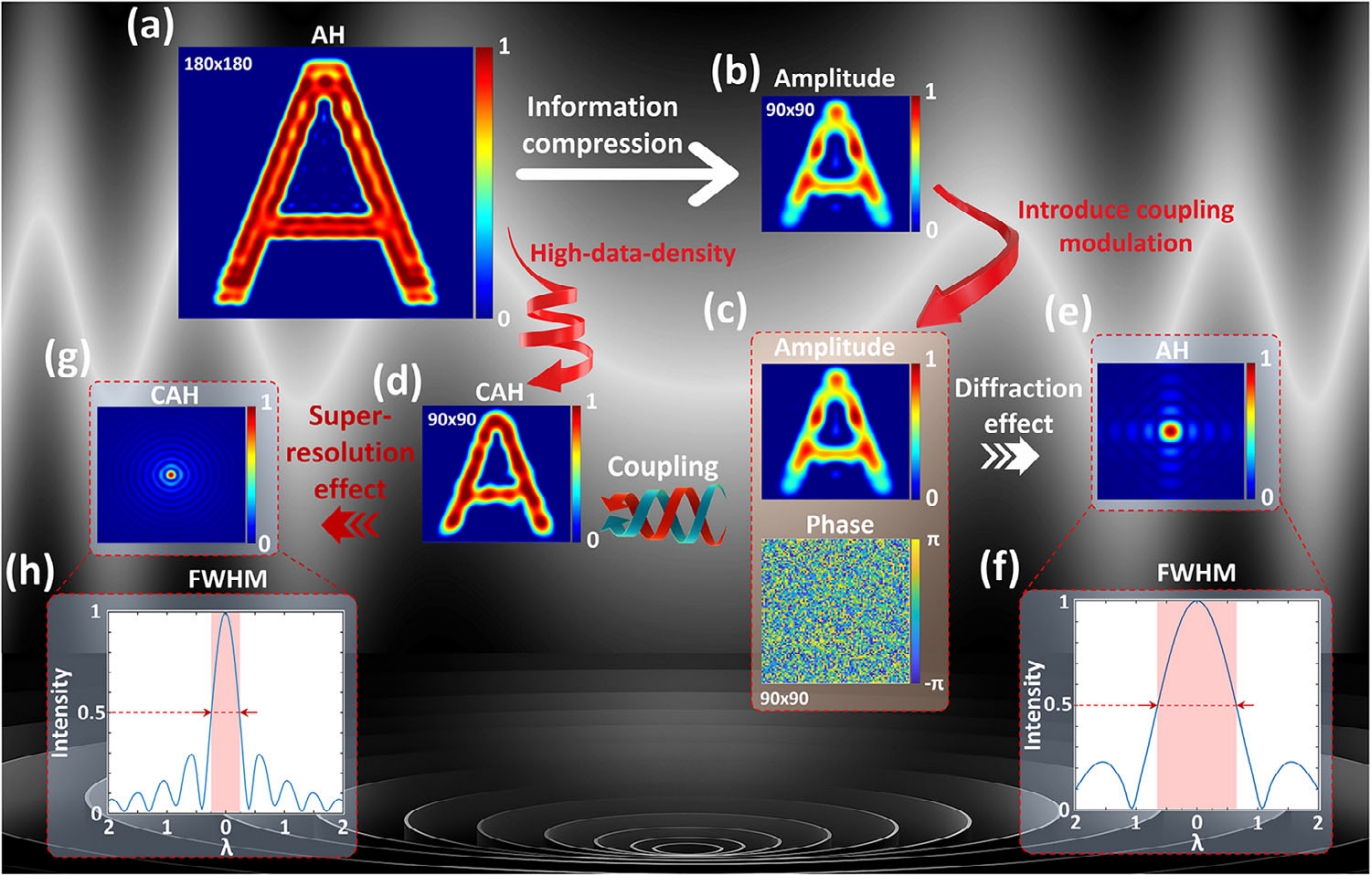
Super‐resolution and high‐data‐density acoustic hologram. (a) High‐data‐density hologram of the letter A with a bandwidth product of 180 × 180 pixels. (b) Low‐data‐density hologram of the letter A with information compressed to a bandwidth product of 90 × 90 pixels. (c) Phase‐coupled modulation achieves super‐resolution holograms. (d) Coupling amplitude hologram (CAH). (e) Single‐pixel hologram. (f) Full width at half maximum (FWHM) of the single‐pixel hologram. (g) Single‐pixel super resolution hologram. (h) FWHM of the single‐pixel CAH.

In the field of acoustic holography, the data density or information capacity of a hologram at the physical level is strictly quantified as the scalar space‐bandwidth product (SBP), which represents the total number of independently addressable pixel units on the holographic plane. The core approach to improving image resolution typically involves increasing the space‐bandwidth product or raising the spatial sampling rate to achieve finer image reconstruction. Previous studies have shown that amplitude‐phase modulation (APM) offers greater freedom in wavefield control compared to pure phase modulation (PM), thereby significantly improving the quality of reconstructed images [17]. However, given practical hardware constraints, encoding more high‐frequency spatial information onto physical arrays with low SBP to achieve high‐resolution imaging remains a significant challenge in the field of acoustic holography. Therefore, this work focuses on enhancing image resolution and data density within a limited SBP by investigating the physical mechanisms of acoustic holographic imaging. Using the letter “A” as the imaging target, we systematically compare the reconstruction qualities of the amplitude hologram (AH) under different pixel densities using APM modulation. This comparison is based on a high‐data‐density hologram (Figure 1a) with 180 × 180 pixels, from which a low‐data‐density sample (Figure 1b) is constructed via information compression.

We focus on investigating the amplitude‐phase coupling modulation mechanism. Based on the Helmholtz equation governing acoustic wave propagation, we establish the coupling relationship between complex sound pressure amplitude and phase through the sound pressure equation $P\;\left(x\right)=A\left(x\right)e^{i\phi (x)}$ and the wave equation $\frac{d^{2}P\left(x\right)}{dx^{2}}+k^{2}P\;\left(x\right)=0$.

(3) $$\begin{array}{cc} \frac{d^{2}A\left(x\right)}{dx^{2}}+ & 2i\frac{dA\left(x\right)}{dx}\frac{d\phi \left(x\right)}{dx}+iA\left(x\right)\frac{d^{2}\phi \left(x\right)}{dx^{2}}\\ & -A\left(x\right){\left(\frac{d\phi \left(x\right)}{dx}\right)}^{2}+k^{2}A\;\left(x\right)=0 \end{array}$$

The differential properties of sound pressure reveal the physical nature of coupling between amplitude and phase. The wave equation for complex sound pressure can be decomposed into the real part $\frac{d^{2}A\left(x\right)}{dx^{2}}$ governing amplitude evolution and the imaginary part $\frac{d^{2}\phi \left(x\right)}{dx^{2}}$ governing the dominant dynamic phase‐amplitude coupling (See Figure S1 in Note S1 for the coupling mechanism of amplitude and phase).

(4) $$\left\{\begin{array}{c} \;\frac{d^{2}A\left(x\right)}{dx^{2}}=A\left(x\right)\left({\left(\frac{d\phi \left(x\right)}{dx}\right)}^{2}-k^{2}\right)\\ \;\frac{d^{2}\phi \left(x\right)}{dx^{2}}=-\frac{2}{A\left(x\right)}\frac{dA\left(x\right)}{dx}\frac{d\phi \left(x\right)}{dx} \end{array}\right.$$

Under plane wave excitation conditions, the sound field amplitude remains constant (*A* (*x*) = *A*_0_), so the real part of the wave equation governing amplitude‐phase coupling is constant. Simultaneously, the phase varies linearly ($\phi \left(x\right)=kx+\phi_{0}$). At this point, the imaginary part equation governs the dynamic coupling behavior between amplitude and phase. Through mathematical transformation, the imaginary part of this coupling term can be rewritten as $\frac{d}{dx}\;\left(A^{2}\frac{d\phi }{dx}\right)=0$. This equation indicates that the acoustic energy remains constant in the propagation direction, thus enabling the establishment of a relationship between amplitude‐phase coupling and energy conservation.

(5) $$J\;\left(x\right)=A^{2}\frac{d\phi }{dx}$$

The above reveals the dynamic coupling relationship between amplitude *A*(x) and phase *ϕ*(x). Based on this energy conservation constraint, we achieve efficient amplitude‐phase coupling modulation by regulating the energy conservation relationship between amplitude and phase.

Under plane wave conditions with an imaging distance of 20 cm and a frequency of 17 kHz, we introduce the coupled focusing phase distribution of the AH (Figure 1c). Through reversal, we obtain the phase distribution that enhances the focusing effect of the AH. By presetting the reversal phase as the initial phase of the AH and utilizing the amplitude‐phase coupled acoustic energy conservation modulation mechanism, we achieve high‐resolution acoustic field focusing of the AH under low data density conditions. As shown in Figure 1d, this method generates a super resolution hologram.

To further validate the effectiveness of the proposed method, we compared the traditional AH of a single pixel (Figure 1e) with the FWHM curve distribution (Figure 1f), as well as the CAH of a single pixel (Figure 1g) with the FWHM curve distribution (Figure 1h).

### 2.2 Metasurface Sample Design Method and Experimental Setup

**FIGURE 2:**
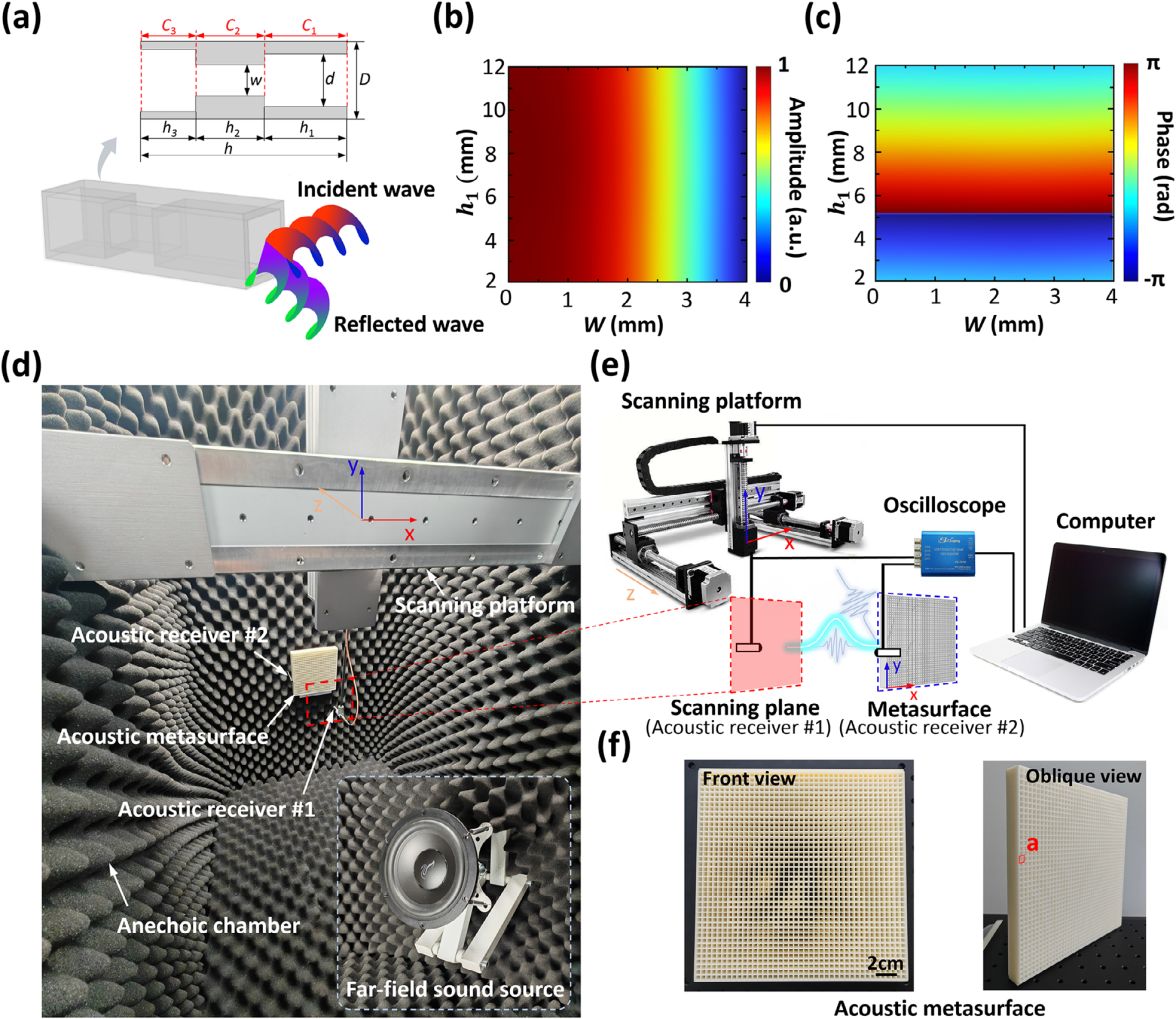
Metasurface design method and experimental setup. (a) Unit cell structure in metasurface. (b, c) Reflection amplitude and phase response of cells to parameters h1 and w under decoupling conditions. (d) Scanning the experimental platform. (e) Field sweeping platform control system. (f) Front and oblique views of the acoustic metasurface sample.

We have fabricated an acoustic metasurface sample integrating amplitude and phase focusing using 3D printing technology with a precision of 0.1 mm, whose structure is shown in Figure 2a. This metasurface unit comprises three parallel channels (*C*_1_, *C*_2_, *C*_3_) with corresponding heights *h*_1_, *h*_2_, and *h*_3_. The widths of channels *C*_1_ and *C*_3_ are set to $d=\beta \cdot \frac{\lambda }{4}$, where β  =  0.8 represents the filling ratio, and *λ* denotes the wavelength of sound waves in air. The total width of the unit is *d*. The width w of the middle channel *C*_2_ is an adjustable parameter, with the channel walls treated as acoustically rigid boundaries and *h*_2_ fixed at 5 mm. By adjusting the height *h*_1_ of the incident channel and the width w of the middle channel, independent control of the reflected sound wave's amplitude and phase can be achieved. This enables continuous control of amplitude within the range [0,1] and phase within the range [‐π, π] on the holographic plane [17]. Figure 2b,c illustrates the response relationships of the unit's reflected amplitude and phase as functions of *h*_1_ and *w*, verifying the structure's amplitude‐phase decoupling capability under vertical incidence conditions (See Figure S2 in Note S2 for hologram metasurface design). The mapping relationship between *h*_1_, *w*, and the reflected coefficient's amplitude and phase can be characterized by the following expressions based on the acoustic propagation model.

(6) $$A=\frac{d^{4}-w^{4}}{d^{4}+w^{4}}\;$$

(7) $$\phi =-\frac{4\pi h_{1}}{\lambda }$$

The experimental measurement system comprised a three‐axis motion platform with 0.05 mm precision, serving as the acoustic field scanning device. Acoustic receiver #1 was fixed to the moving end of the motion platform, tracking its movement to collect the reflected acoustic field modulated by the metasurface. Acoustic receiver #2 was fixed at the same plane position on the metasurface to collect the incident acoustic field at that location, with a separation of 20 cm between the two receivers.

During the scanning process, the *z*‐axis of the motion platform adjusts the imaging distance. The X‐Y plane is parallel to the metasurface plane, and the moving end of the motion platform drives acoustic receiver #1 to perform a two‐dimensional scan of the modulated acoustic field. The output signals from the two microphones are connected to two channels of the oscilloscope. Control of the oscilloscope, motion platform, and far‐field sound source is integrated within the LabVIEW system. The system pauses the motion platform for 2 s after every 2 mm of movement. During this interval, the far‐field sound source triggers the emission of a pulse sound signal. Simultaneously, the system acquires the time‐domain signal of the reflected sound field received by receiver #1 and the time‐domain signal of the incident sound field received by receiver #2. This pulse operation mode effectively avoids reverberation interference caused by continuous sound fields, thereby enhancing the signal‐to‐noise ratio and accuracy of sound field reconstruction.

### 2.3 Experimental Demonstration of Super‐Resolution Imaging

**FIGURE 3:**
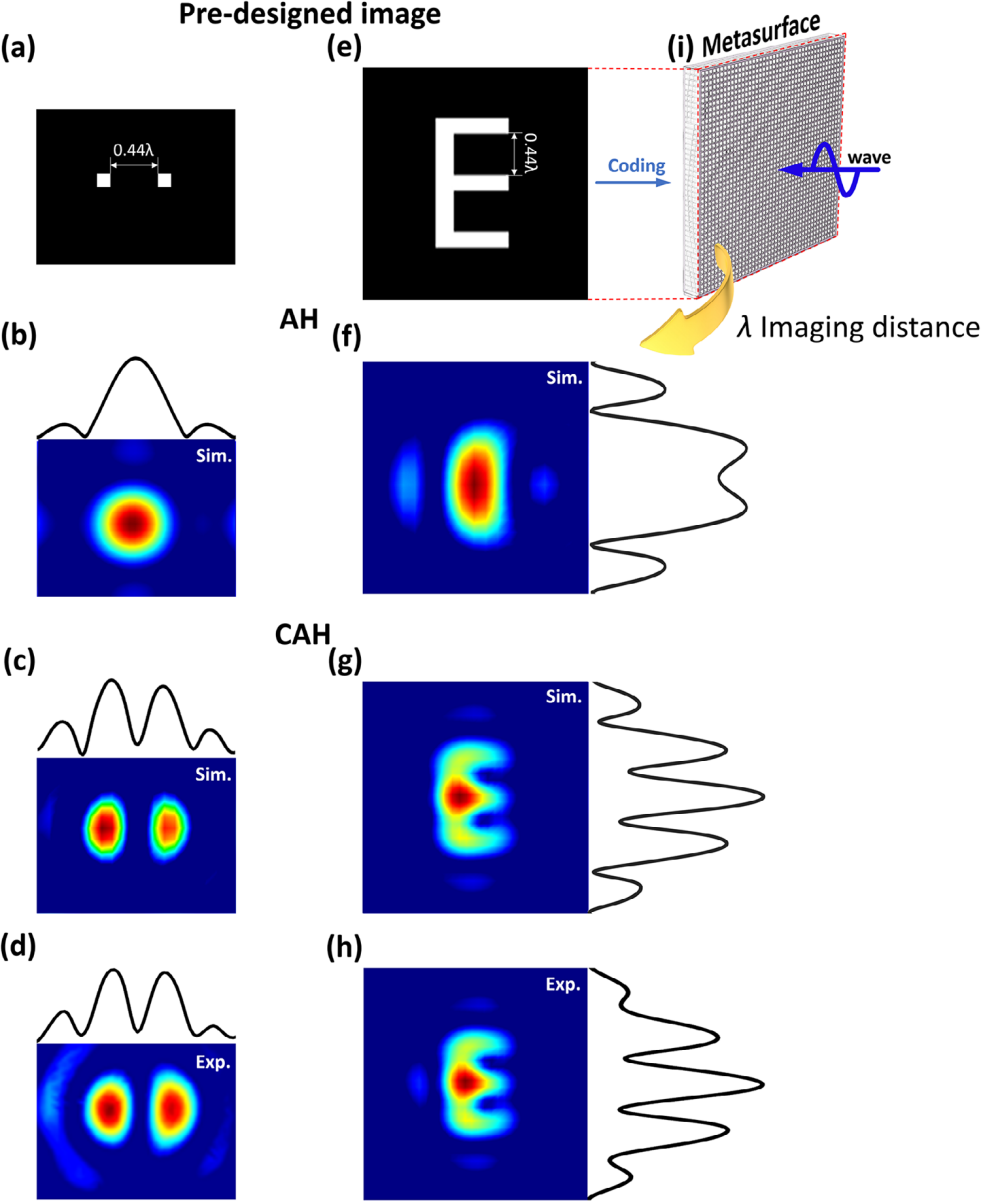
Experiment demonstration of super‐resolution imaging. (a) Pre‐designed image with pixel spacing of 0.44*λ*. (b) Hologram with pixel spacing of 0.44*λ*. (c) Numerically simulated hologram with pixel spacing of 0.44*λ* after amplitude‐phase coupling modulation. (d) A two‐pixel experimental hologram. (e) Pre‐designed image of letter E with aperture of 0.44*λ*. (f) Hologram of letter E. (g) Coupled‐focusing phase numerically simulated hologram of the letter E. (h) Experimental hologram of the letter E. (i) Metasurface structure with APM modulation capability.

To validate the super‐resolution performance of the proposed focused phase‐modulated hologram at subwavelength scales, we have reconstructed the acoustic field at an imaging distance of *λ* = 2 cm for two pre‐designed images with subwavelength pixels separated by only 0.44*λ* = 0.88 cm, as shown in Figure 3a,e, under excitation by a 17 kHz plane wave. As shown in Figure 3b, the conventional APM hologram cannot resolve this subwavelength spacing due to the diffraction limit. Sound waves cannot effectively transmit through the region between the two pixels, resulting in the reconstructed acoustic field merging into a single focal spot with a single peak in the sound pressure distribution. In contrast, the proposed hologram coupling a focusing phase distribution achieves clear resolution as demonstrated by the numerical simulations and experimental results shown in Figure 3c,d, respectively. Sound waves penetrate gaps smaller than half a wavelength, forming two distinct focal spots with two clearly separated main peaks in the sound pressure distribution. Furthermore, we conducted further experiments to determine that the effective super‐resolution range of CAH under far‐field conditions is 0.25λ to 2.5λ, and quantitatively revealed that its spatial resolution exhibits a nonlinear decay pattern with increasing propagation distance (See Figure S3 in Note S3 for the analysis of CAH attenuation characteristics in far‐field conditions and quantitative evaluation of holographic imaging efficiency). Experimental results demonstrate that CAH effectively overcomes the diffraction limit of sound waves, significantly enhancing hologram quality through the acoustic focusing effect.

To further validate the versatility and super‐resolution imaging capabilities of this method, this study selected letter patterns with sharp boundaries and deep subwavelength gaps as test targets for super‐resolution imaging. Compared to complex patterns that tend to mask edge step responses, this approach enables the precise extraction of the FWHM of the acoustic field, thereby allowing for a rigorous and quantitative assessment of the system's actual focusing performance beyond the acoustic diffraction limit. Based on this, we selected the letter “E” with an internal aperture smaller than half a wavelength as the target image and used the APM method to modulate the amplitude and phase of the incident sound waves. The AH experimental results exhibited diffraction effects, failing to recognize subwavelength details within the internal structure of the letter “E,” as shown in Figure 3f. In contrast, the CAH experimental results faithfully reconstructed the internal detail contours of the letter “E,” rendering its overall subwavelength aperture structure clearly visible, as demonstrated in Figure 3g,h.

The mechanism of CAH achieving super‐resolution holographic imaging involves coupling phase gradient distributions with acoustic focusing effects to enable effective acoustic focusing modulation. Specifically, scattered acoustic waves generated by diffraction effects within the holographic acoustic field strongly couple with the phase distribution obtained through inversion calculations. This coupling re‐focuses the scattered acoustic energy, enabling high‐resolution acoustic field reconstruction at subwavelength scales. The peak distribution of the acoustic energy FWHM curves shown on the right side of Figure 3f–h further validates this conclusion. After coupling with the focusing phase, the hologram exhibits three distinct subwavelength‐scale energy peaks, closely matching the letter “E” shape as depicted in Figure 3g,h. In contrast, conventional methods only excite two weaker, diffusely distributed peaks, as shown in Figure 3f. While achieving the aforementioned high spatial resolution reconstruction, we further conducted a quantitative decoupling evaluation of the experimental system's energy transfer performance. The data indicate that the reflection efficiency of this amplitude‐phase‐coupled modulation mechanism at the metasurface is 35.03%, while the spatial focusing efficiency in the target acoustic field region is 45.06%; the total holographic imaging efficiency of the system is thus calculated to be 15.79% (See Figure S4 in Note S3 for the analysis of CAH attenuation characteristics in far‐field conditions and quantitative evaluation of holographic imaging efficiency). The results demonstrate that the proposed amplitude‐phase coupling of holograms not only overcomes the diffraction limit but also enables high‐resolution manipulation of far‐field acoustic waves at the wavelength scale. This approach overcomes the range limitations of conventional near‐field evanescent wave methods, providing a new physical foundation and advanced conceptual technical pathway for high‐resolution acoustic information transmission and imaging in the far field.

### 2.4 High‐Data‐Density Hologram of CAH

**FIGURE 4:**
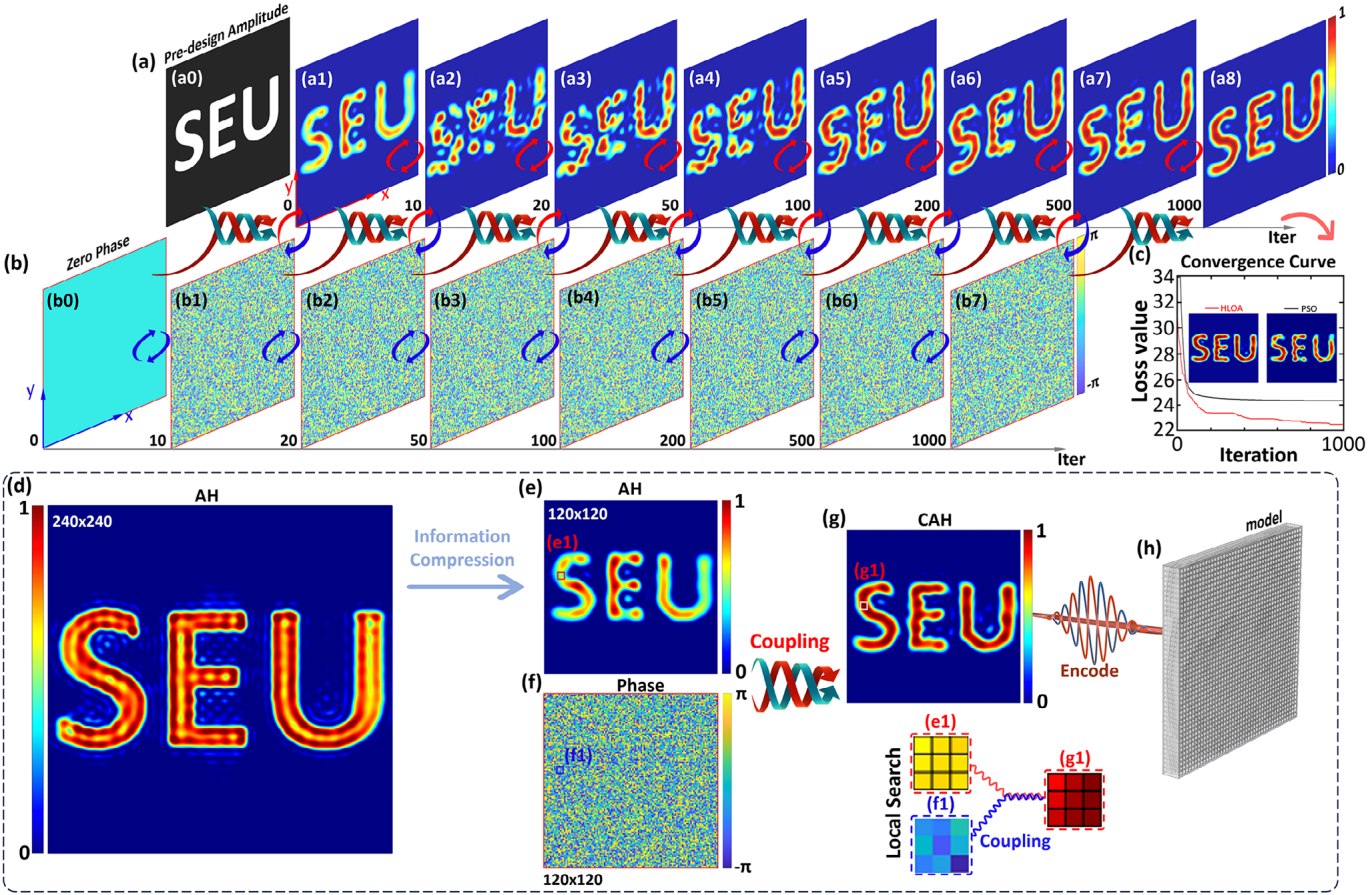
High data density hologram of CAH. (a) Amplitude hologram (AH) (A0 pre‐designed amplitude. A1‐A8 dynamic response of hologram to different phase distributions). (b) Focused phase distributions corresponding to different holograms. (c) Convergence curve. (d) Hologram with 240 × 240 image pixel bandwidth product. (e) Hologram with information compressed to 120 × 120 image pixel bandwidth product (e1 Low‐resolution holographic pixel region). (f) Coupled focusing phase distribution corresponding to the 120 × 120 image pixel bandwidth product hologram (f1 Focusing phase distribution corresponding to the low‐resolution holographic pixel region). (g) CAH (g1 Local pixel distribution of CAH). (h) Three‐dimensional model of acoustic metasurface encoding amplitude and phase of CAH.

To validate high‐resolution holographic imaging capabilities for complex patterns under low information density conditions, the multi‐letter “SEU” was selected as the target image. This image was discretized and placed within an image plane defined by a 120 × 120 pixel SBP. Significant multidimensional coupling effects exist between amplitude and phase in the hologram. We have constructed a coupled amplitude‐phase mathematical model, derived the evolution law of amplitude with phase from physical principles, and subsequently performed reversal to obtain the optimal focusing phase distribution of the hologram. Figure 4 illustrates the process of performing reversal of the coupled focusing phase distribution corresponding to the high‐resolution hologram using HLOA, based on the principle of acoustic energy conservation. (See Note S4 for the physical mechanism of HLOA solving focusing phase distribution based on time‐reversal principle).

Figure 4a demonstrates the hologram's distinct dynamic response to different focusing phase distributions, with this high‐resolution sound field reconstruction mechanism originating from the effective regulation of the wavefront by the acoustic phase gradient. Figure 4b displays the focusing phase distribution during the reversal process. Figure 4c shows the convergence curves for the HLOA and particle swarm optimization (PSO) [36, 37] reversal processes, along with the corresponding holographic imaging results. (See Figure S5 in Note S4 for the physical mechanism of HLOA solving focusing phase distribution based on time‐reversal principle). During this process, the wavefront diffracted by the hologram strongly couples with the introduced focusing phase. Through the phase‐focusing effect of CAH, discrete acoustic energy is focused and reconstructed under low information density conditions, enabling high‐resolution hologram reconstruction. This method holds promise for overcoming diffraction‐limited constraints in far‐field acoustic environments, offering a novel technical pathway to enhance data density in communications. Figure 4d–i demonstrates the validation of 4 times information compression process for a 240 × 240 high‐information‐density hologram, yielding a 120 × 120 low‐information‐density hologram. The process of reconstructing a high‐resolution hologram under low information density by reversal of the coupled focusing phase distribution based on the conservation of acoustic energy is shown in Figure 4g. To further demonstrate the local amplitude‐phase coupling effect, we have extracted nine low‐resolution pixel regions from the AH after information compression (as shown in Figure 4e, 1) and their corresponding phase distributions in the reversal phase map (as shown in Figure 4f, 1). The results demonstrate that local amplitude‐phase coupling effectively achieves high‐resolution focusing, as shown in Figure 4g, 1. Based on this design, we have constructed a three‐dimensional model of an acoustic metasurface that encodes the amplitude and phase of CAH, as shown in Figure 4h.

### 2.5 Experimental Demonstration of High‐Data‐Density Acoustic Hologram

**FIGURE 5:**
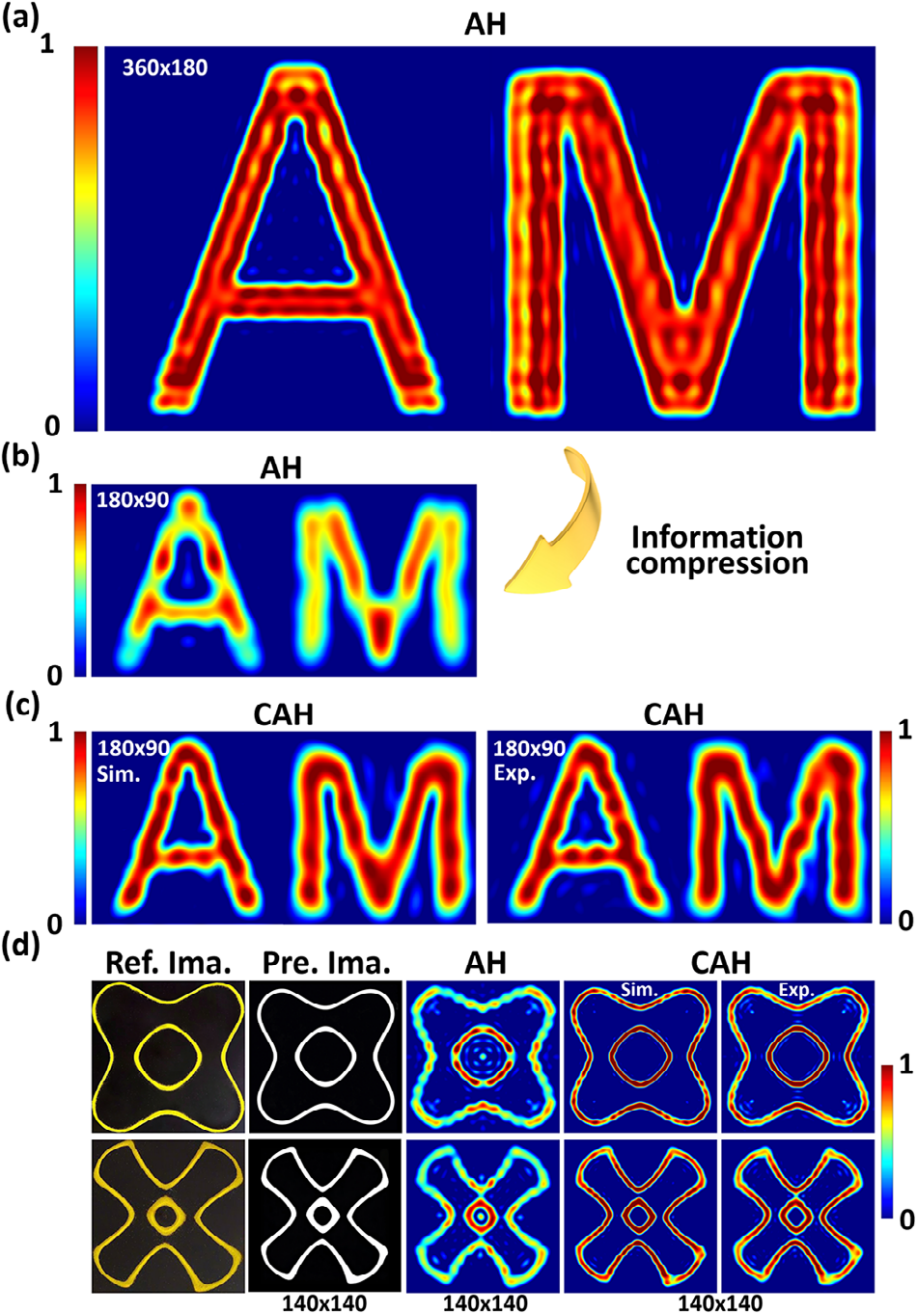
Experiment demonstration of high‐data‐density acoustic hologram. (a) Hologram under high SBP conditions (360 × 180). (b) Hologram under low SBP conditions (180 × 90). (c) Numerical simulation and experimental results of CAH obtained by introducing a coupled focusing phase distribution under the same low SBP conditions. (d) High‐data‐density reconstruction of the core intersection region of a Chladni nodal pattern at a 140 × 140 SBP, including the real‐world reference image, pre‐designed image, and comparisons between AH and CAH.

The resolution of acoustic holograms is largely determined by their SBP. Typically, a larger SBP corresponds to higher information density, thereby facilitating higher‐resolution holographic reconstruction. However, constrained by the diffraction limit of sound waves, achieving high‐resolution imaging with holograms remains significantly challenging under low information density conditions. To validate the effectiveness of CAH in enhancing hologram resolution under low data density conditions, Figure 5 presents numerical simulations and experimental results using the letter “AM” as the target image. The first row displays the AH of the letter “AM” at 360 × 180 high SBP. The second row shows the low‐resolution AH of the letter “AM” after compressing the SBP to 180 × 90. The third row presents numerical simulations and experimental results of CAH under the same low SBP conditions. Experimental findings demonstrate that under low SBP conditions, CAH's effective wavefront phase‐focusing modulation can significantly enhance the resolution of reconstructed holograms, thereby effectively overcoming the diffraction limit's constraints on imaging quality.

Figure 5 shows the experimental results of AH and CAH when the SBP is reduced from 360 × 180 to 180 × 90. The results indicate that data density severely limits hologram resolution. However, by introducing coupled‐focusing phase modulation, CAH achieves high‐resolution holographic imaging characteristics while exhibiting higher acoustic pressure amplitude distribution in the letter region. The experimental results demonstrate that the CAH with coupled focusing phase modulation overcomes the acoustic diffraction limit at subwavelength scales, achieving high‐precision wavefront modulation and high‐fidelity acoustic field reconstruction. These findings conclusively validate that introducing a focusing phase distribution can effectively enable super‐resolution holographic acoustic field reconstruction at low data density, opening a novel pathway for high‐dimensional information encoding and data transmission in far‐field acoustic communication.

To further validate the CAH mechanism's ability to process highly complex holographic information with limited physical data density, this study introduced a local feature region of the Crani spectrum representing acoustic resonance eigenmodes as a test target, as shown in Figure 5d. At a low SBP of 140 × 140, this target pattern exhibits multiple continuous curves that closely approach and intersect one another, placing more stringent demands on the global phase coherence of the wavefield. Compared to the severe diffraction aliasing generated by traditional AH methods at the intersection nodes, both the numerical simulations and physical experimental results of CAH clearly reconstructed the complete continuous curve contours, effectively suppressing background crosstalk. In addition, this study conducted a validation study on complex butterfly and clover patterns characterized by multiscale continuous curves (See Figure S6 in Note S5 for the High‐data‐density holographic reconstruction of complex topological patterns). These results further confirm the system's ability to process complex features at high spatial frequencies under constrained physical arrays.

Experimental reconstruction results closely match numerical simulations in the overall hologram profile, though edge curvature and distortion persist in localized regions. This discrepancy stems partly from environmental noise interference and partly from limitations in the manufacturing precision of the holographic metasurface. The current 3D printing process employed has constraints regarding the geometric dimensions of unit cells and the accuracy of phase distribution control. Therefore, further enhancing the processing precision and structural fidelity of the metasurface will facilitate clearer and more precise holographic sound field reconstruction.

### 2.6 Quantitative Analysis of Far‐Field and High‐Data‐Density Information Transmission

The resolution of acoustic holograms is primarily constrained by the combined effects of acoustic wave propagation characteristics and wavelength *λ*. This study has successfully demonstrated the resolution capability of CAH at the subwavelength scale, surpassing the conventional *λ*/2 diffraction limit. Building upon this foundation, we further evaluate its far‐field imaging performance. While conventional evanescent waves enable super‐resolution imaging within the near‐field range below the wavelength *λ*, their imaging distance is constrained by attenuation characteristics, making it difficult to overcome the limitations of far‐field super‐resolution imaging beyond the wavelength scale. Therefore, building upon the breakthrough of the diffraction limit at the wavelength scale, we extended the imaging distance and reduced the SBP to investigate the imaging mechanism and performance limits of CAH under far‐field conditions and low data density. This approach aims to break the limitations of holograms in far‐field high‐resolution acoustic imaging and communication applications.

**FIGURE 6:**
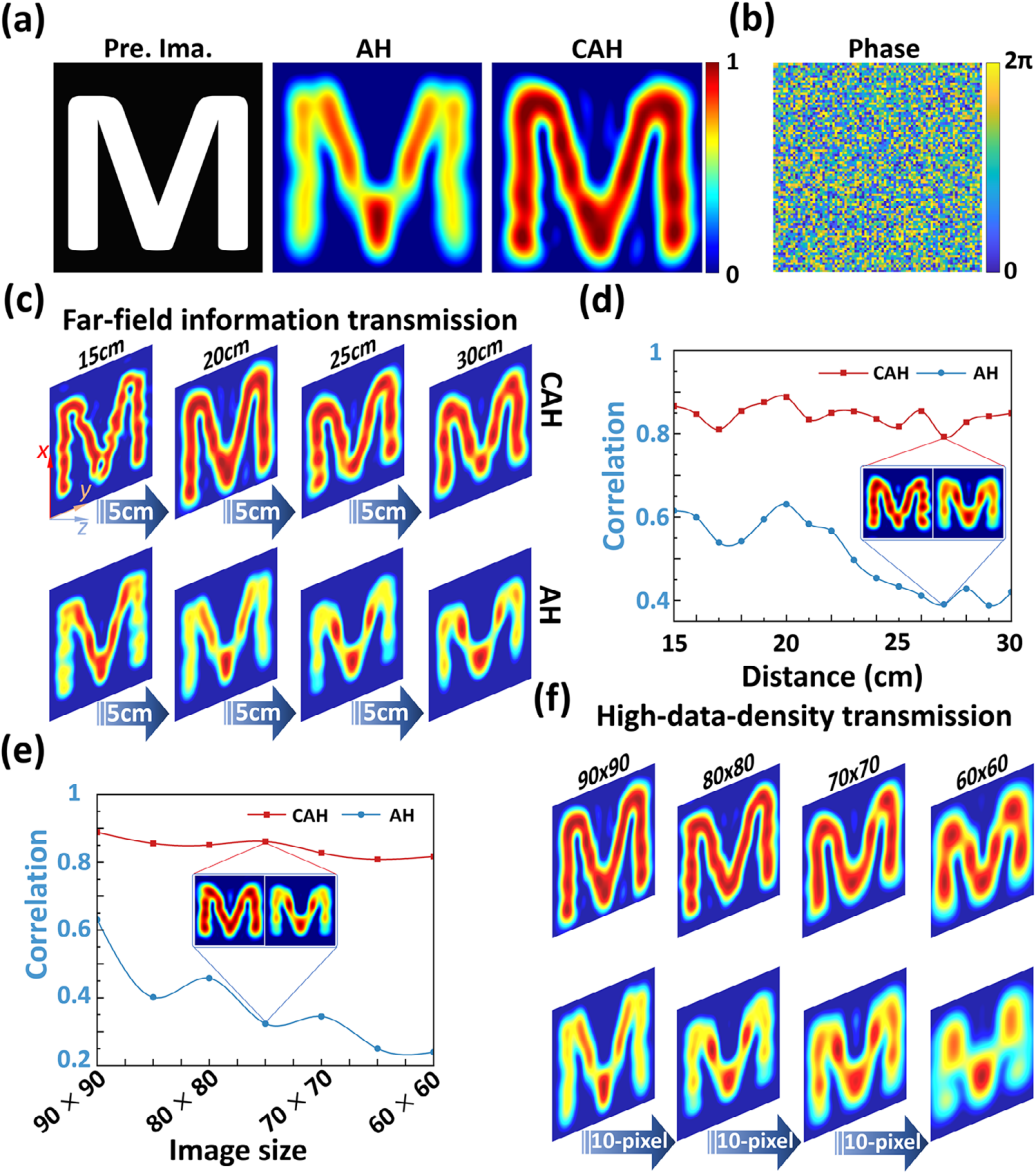
Quantitative analysis of far‐field and high‐data‐density information transmission. (a) The reconstruction results of the pre‐designed image, AH, and CAH. (b) Focusing phase distribution. (c) Compare the reconstructed images of CAH and AH at 5 cm intervals. (d) Correlation performance evaluation of CAH and AH within the 15–25 cm far‐field imaging distance range. (e) Correlation coefficient curves for CAH and AH methods at different information densities. (f) Visual comparison of holograms displayed at 10‐pixel intervals.

The main body of this study uses the second letter “M” of the amplitude, featuring sharp‐edged characteristics, as a resolution test target to quantitatively evaluate the system's diffraction‐limited physical focusing performance through edge response analysis. However, the information capacity of an acoustic holographic system is limited by the SBP. Specifically, the maximum degree of freedom for wavefield control in a metasurface composed of *N* × *N* subwavelength units is *N* ^2^. Figure 6a sequentially displays the pre‐designed images, AH, and CAH under 90 × 90 pixel SBP. Figure 6b shows the focused phase distribution of CAH. Figure 6c presents reconstructed holograms of CAH and AH at a 5 cm spacing. Results indicate that CAH exhibits superior robustness compared to AH as the imaging distance increases. The acoustic field distribution within the CAH letter region maintains high resolution and edge sharpness. In contrast, AH's image resolution degrades sharply with increasing distance due to acoustic diffraction effects. Figure 6d quantitatively evaluates image correlation between CAH and AH across the 15–25 cm far‐field imaging range (See Figure S7 in Note S6 for the Correlation and NMSE characterization of image quality). Results indicate that within the 15–25 cm imaging range, the CAH image correlation curve exhibits a smoother decline, consistently maintaining above 80% and peaking near 90% at approximately 20 cm. In contrast, the AH image correlation is markedly lower than CAH, with its correlation curve showing a steep downward trend.

To study the impact of data density on holographic imaging quality, we compared the hologram correlation between AH and CAH within the 90 × 90 to 60 × 60 pixel range at a 20 cm imaging distance. Figure 6e displays the correlation curves of AH and CAH at different SBP levels. Within this SBP range, CAH demonstrated significantly superior image correlation compared to AH. The CAH correlation curve exhibited a slower decline with decreasing SBP than AH, maintaining values above 80% across all SBP ranges. This indicates CAH possesses higher robustness under low data density conditions. Figure 6f further displays holograms at different pixel SBP values at 10‐pixel intervals. When SBP decreased to 60 × 60, AH exhibited severe image quality degradation due to reduced data density, whereas CAH maintained relatively clear contours and stronger sound pressure distribution. Experimental results demonstrate that image data density critically impacts hologram imaging quality, while highlighting the dual advantages of the proposed CAH in enhancing image resolution and enabling high‐density information transmission.

These results demonstrate that CAH possesses superior acoustic field transmission and reconstruction capabilities, enabling high‐resolution acoustic imaging under low data density conditions.

## 3 Conclusion

This research has introduced an innovative concept for constructing acoustic holograms based on amplitude‐phase coupling modulation, successfully overcoming the diffraction limit of sound waves at the wavelength scale and achieving a super‐resolution acoustic field reconstruction under low information density conditions. Through theoretical derivation of the differential characteristics of sound pressure, we reveal the relationship between amplitude‐phase coupling and acoustic energy conservation. We further invert the coupled focusing phase distribution of the hologram, which significantly enhances the focusing performance of the hologram. In validation experiments, using both a double‐point model with spacing less than half a wavelength and a letter “E” model with subwavelength apertures, the CAH clearly resolved fine structures inaccessible to conventional methods. Its transmission focal length significantly outperforms conventional near‐field range evanescent wave super‐resolution imaging techniques, which are far shorter than the wavelength. Furthermore, under low SBP conditions, complex patterns with “SEU” as the target achieved high‐resolution reconstruction at a far‐field imaging distance of 20 cm, demonstrating the method's capability for high‐resolution acoustic field transmission even under low information density conditions. This research offers novel insights and approaches for far‐field acoustic super‐resolution imaging and low‐density information transmission, holding significant application potential in fields such as medical ultrasound imaging and long‐range acoustic communication.

Furthermore, the core logic of solving the inverse problem based on the scalar wave equation exhibits strong interdisciplinary universality. Therefore, the proposed amplitude‐phase coupling mathematical model and the introduced HLOA framework hold substantial potential for extension into the electromagnetic (optical) domain. However, extending this scheme to achieve optical super‐resolution requires addressing fundamental physical distinctions at the subwavelength scale. Specifically, the underlying propagation kernel must be comprehensively upgraded from a scalar acoustic model to a full‐vectorial electromagnetic model to rigorously account for the intense near‐field vectorial coupling, polarization cross‐talk, and complex resonance mechanisms inherent to optical metasurfaces. We envision this cross‐disciplinary adaptation and the resolution of these physical challenges as a highly promising avenue for future work.

## 4 Experimental Section

### 4.1 Numerical Simulations

Numerical simulations were performed using MATLAB 2025a, in which a time‐reversal based combined with the HLOA algorithm was employed to inversely solve the focusing phase distribution under the constraint of acoustic energy conservation. The resulting amplitude and phase profiles on the hologram plane obtained from MATLAB were subsequently imported into COMSOL Multiphysics 6.2. A three‐dimensional finite element model of the acoustic metasurface was constructed, and the pressure acoustic module was used to compute the resultant sound field.

### 4.2 Experimental Setup

The experimental equipment is shown in Figure 2. We used a 3D printer with a printing precision of 0.1 mm to print acoustic metasurface samples made of photopolymer resin. The photopolymer resin material has a density of 1.13 g/cm^3^, a Young's modulus of 2642 MPa, and a Poisson's ratio of 0.42. Acoustic absorption cotton was arranged inside the experimental equipment to prevent the influence of reflected sound waves. Two identical microphones (BSWA MPA416) were connected to a data acquisition card (VK‐701H). A three‐axis positioning stage with a motion accuracy of 0.05 mm was employed to control the scanning trajectory. A sound source operating at 17 kHz, corresponding to an acoustic wavelength of 2 cm, was used for wave excitation. All components were integrated and synchronously controlled through a LabVIEW‐based system.

## Author Contributions

X.G. performed the theoretical simulations. X.G., X.Z.W., G.S.T., H.H.Z., S.Q.F., Y.Y.M., Z.Y.H., T.T.L., H.X., J.S., and H.Y.F. designed and conducted the experiments. X.G., Y.F.Z., H.Z., and B.A. wrote the manuscript. X.X.G., Y.F.Z., H.Z., and B.A. guided the research. All other authors contributed to data analysis and discussions.

## Conflicts of Interest

The authors declare no conflicts of interest.

## Supporting information




**Supporting File**: advs76251‐sup‐0001‐SuppMat.docx.

## Data Availability

The data that support the findings of this study are available from the corresponding author upon reasonable request.

## References

[1] K. Melde , A. G. Mark , T. Qiu , and P. Fischer , “Holograms for Acoustics,” Nature 537 (2016): 518–522, 10.1038/nature19755. 27652563

[2] D. G. Grier , “A Revolution in Optical Manipulation,” Nature 424 (2003): 810–816, 10.1038/nature01935. 12917694

[3] X.‐B. Miao , H.‐W. Dong , S.‐D. Zhao , et al., “Deep‐Learning‐Aided Metasurface Design for Megapixel Acoustic Hologram,” Applied Physics Reviews 10 (2023): 021411, 10.1063/5.0136802.

[4] K. Wu , J.‐J. Liu , Y. Ding , W. Wang , B. Liang , and J.‐C. Cheng , “Metamaterial‐Based Real‐Time Communication With High Information Density by Multipath Twisting of Acoustic Wave,” Nature Communications 13 (2022): 5171, 10.1038/s41467-022-32778-z. PMC9440107 36055988

[5] J. W. Goodman , Introduction to Fourier Optics (Roberts and Company publishers, 2005).

[6] X. Zhou , M. B. Assouar , and M. Oudich , “Acoustic Superfocusing by Solid Phononic Crystals,” Applied Physics Letters 105 (2014): 233506, 10.1063/1.4904262.

[7] Y. Zhu , L. Cao , A. Merkel , S.‐W. Fan , and B. Assouar , “Bifunctional Superlens for Simultaneous Flexural and Acoustic Wave Superfocusing,” Applied Physics Letters 116 (2020): 253502, 10.1063/5.0004428.

[8] G. Ma and P. Sheng , “Acoustic Metamaterials: from Local Resonances to Broad Horizons,” Science Advances 2 (2016): 1501595. 10.1126/sciadv.1501595 PMC4771441 26933692

[9] H. Ge , M. Yang , C. Ma , et al., “Breaking the Barriers: Advances in Acoustic Functional Materials,” National Science Review 5 (2018): 159–182, 10.1093/nsr/nwx154.

[10] S. A. Cummer , J. Christensen , and A. Alù , “Controlling Sound With Acoustic Metamaterials,” Nature Reviews Materials 1 (2016): 16001, 10.1038/natrevmats.2016.1.

[11] B. Assouar , B. Liang , Y. Wu , Y. Li , J.‐C. Cheng , and Y. Jing , “Acoustic Metasurfaces,” Nature Reviews Materials 3 (2018): 460–472, 10.1038/s41578-018-0061-4.

[12] Y. Tian , Q. Wei , Y. Cheng , and X. Liu , “Acoustic Holography Based on Composite Metasurface With Decoupled Modulation of Phase and Amplitude,” Applied Physics Letters 110 (2017): 191901, 10.1063/1.4983282.

[13] A.‐L. Chen , Y.‐S. Wang , Y.‐F. Wang , H.‐T. Zhou , and S.‐M. Yuan , “Design of Acoustic/Elastic Phase Gradient Metasurfaces: Principles, Functional Elements, Tunability, and Coding,” Applied Mechanics Reviews 74 (2022): 020801, 10.1115/1.4054629.

[14] M. D. Brown , “Phase and Amplitude Modulation With Acoustic Holograms,” Applied Physics Letters 115 (2019): 053701, 10.1063/1.5110673.

[15] Y. Zhu , N. J. Gerard , X. Xia , et al., “Systematic Design and Experimental Demonstration of Transmission‐Type Multiplexed Acoustic Metaholograms,” Advanced Functional Materials 31 (2021): 2101947, 10.1002/adfm.202101947.

[16] Y. Zhu , J. Hu , X. Fan , et al., “Fine Manipulation of Sound via Lossy Metamaterials With Independent and Arbitrary Reflection Amplitude and Phase,” Nature Communications 9 (2018): 1632, 10.1038/s41467-018-04103-0. PMC5915438 29691413

[17] A. Chen , Y. Xia , Z. Chen , et al., “Dynamic and High‐Precision Sound Manipulation by Acoustic Metamaterial‐Empowered Phased Arrays,” Advanced Functional Materials 35 (2025): 2425833, 10.1002/adfm.202425833.

[18] Y. Fu , Y. Tian , X. Li , et al., “Asymmetric Generation of Acoustic Vortex Using Dual‐Layer Metasurfaces,” Physical Review Letters 128 (2022): 104501, 10.1103/PhysRevLett.128.104501. 35333072

[19] J. Quan , B. Sun , F. Wang , et al., “Topological‐Charge Multiplexed Metasurfaces for Generating Structural Acoustic Field and Remote Dynamic Control,” Science Advances 11 (2025): adw1701, 10.1126/sciadv.adw1701. PMC12239936 40632843

[20] S. Gao , Y. Li , C. Ma , Y. Cheng , and X. Liu , “Emitting Long‐Distance Spiral Airborne Sound Using Low‐Profile Planar Acoustic Antenna,” Nature Communications 12 (2021): 2006. 10.1038/s41467-021-22325-7 PMC8012347 33790285

[21] Z. Song , S. Xie , H. Ding , et al., “Acoustic Vortex in a Waveguide With a Chiral Gradient Sawtooth Metasurface,” Physical Review Applied 21 (2024): 064006, 10.1103/PhysRevApplied.21.064006.

[22] J. Li , A. Crivoi , X. Peng , et al., “Three Dimensional Acoustic Tweezers With Vortex Streaming,” Communications Physics 4 (2021): 113, 10.1038/s42005-021-00617-0.

[23] A. Marzo , S. A. Seah , B. W. Drinkwater , D. R. Sahoo , B. Long , and S. Subramanian , “Holographic Acoustic Elements for Manipulation of Levitated Objects,” Nature Communications 6 (2015): 8661, 10.1038/ncomms9661. PMC4627579 26505138

[24] X. Guo , H. Zeng , X. Hu , et al., “Phase Hologram by Acoustic Metasurfaces,” Small Structures 7 (2025): 202500354.

[25] K. Melde , H. Kremer , M. Shi , et al., “Compact Holographic Sound Fields Enable Rapid One‐Step Assembly of Matter in 3D,” Science Advances 9 (2023): adf6182, 10.1126/sciadv.adf6182. PMC9908023 36753553

[26] M. Derayatifar , M. Habibi , R. Bhat , and M. Packirisamy , “Holographic Direct Sound Printing,” Nature Communications 15 (2024): 6691, 10.1038/s41467-024-50923-8. PMC11303524 39107289

[27] Z. Ma , A. W. Holle , K. Melde , et al., “Acoustic Holographic Cell Patterning in a Biocompatible Hydrogel,” Advanced Materials 32 (2020): 1904181, 10.1002/adma.201904181. 31782570

[28] J. Kim , S. Kasoji , P. G. Durham , and P. A. Dayton , “Acoustic Holograms for Directing Arbitrary Cavitation Patterns,” Applied Physics Letters 118 (2021): 051902, 10.1063/5.0035298.

[29] P. Zheng , Q. Dai , Z. Li , et al., “Metasurface‐Based Key for Computational Imaging Encryption,” Science Advances 7 (2021): abg0363, 10.1126/sciadv.abg0363. PMC8139587 34020956

[30] H. Zeng , Z. He , Y. Wu , et al., “Amplitude‐Phase Dual‐Channel Encrypted Acoustic Meta‐Holograms,” Advanced Functional Materials 34 (2024): 2405132, 10.1002/adfm.202405132.

[31] H. Zeng , Z. He , T. Zhang , et al., “Four‐Channel Amplitude‐Phase‐Janus‐Encrypted Acoustic Meta‐Hologram,” Advanced Functional Materials (2026): e75873, 10.1002/adfm.75873.

[32] J. Zhu , J. Christensen , J. Jung , et al., “A Holey‐Structured Metamaterial for Acoustic Deep‐Subwavelength Imaging,” Nature Physics 7 (2011): 52–55, 10.1038/nphys1804.

[33] Y.‐X. Shen , Y.‐G. Peng , F. Cai , et al., “Ultrasonic Super‐Oscillation Wave‐Packets With an Acoustic Meta‐Lens,” Nature Communications 10 (2019): 3411, 10.1038/s41467-019-11430-3. PMC6667482 31363090

[34] Z. Li , L. Huang , Q. Sun , et al., “3D Acoustic Imaging Hitting the Diffraction Limit via Fully Parameter‐Optimized Meta‐Lens and Frequency‐Domain Reconstruction,” Advanced Materials 37 (2025): 08453, 10.1002/adma.202508453. PMC12592918 40736141

[35] H. Peraza‐Vázquez , A. Peña‐Delgado , M. Merino‐Treviño , A. B. Morales‐Cepeda , and N. Sinha , “A Novel Metaheuristic Inspired by Horned Lizard Defense Tactics,” Artificial Intelligence Review 57 (2024): 59, 10.1007/s10462-023-10653-7.

[36] J. Kennedy and R. Eberhart , Proceedings of ICNN’95 – International Conference on Neural Networks (IEEE, 1995), 1942–1948.

[37] Y. Shi and R. Eberhart , 1998 IEEE International Conference on Evolutionary Computation Proceedings. IEEE World Congress on Computational Intelligence (Cat. No.98TH8360) (IEEE, 1998), 69–73, 10.1109/ICEC.1998.699146.

