# Supplementary material for: Super‐Resolution and High‐Data‐Density Acoustic Meta‐Hologram via Amplitude and Phase Coupling
Source: Adv Sci (Weinh). 2026 Jun 24:e76251. Online ahead of print. doi: 10.1002/advs.76251 (PMC13337118; doi:10.1002/advs.76251)
Supplement: Supplementary file 1 — Supporting File: advs76251‐sup‐0001‐SuppMat.docx. [file ADVS-9999-e76251-s001.docx]

Supporting Information

Super-Resolution and High-Data-Density Acoustic Meta-Hologram via Amplitude and Phase Coupling

Xiao Guo^1^, Xinzong Wang^1^, Guoshen Tang^1^, Haohan Zeng^1^, Siqi Fan^1^, Xinghao Hu^1^, Youyu Mo^1^, Zhenyu He^1^, Tingting Li^1^, Hui Xu^1^, Jiao Shen^1^, Haiyan Fan^1,2^, Xiaoxiang Gao^3,4^*, Yifan Zhu^1,2^*, Hui Zhang^1,2^*, Badreddine Assouar^5^*

Corresponding author:

yifanzhu@seu.edu.cn

seuzhanghui@seu.edu.cn

gaoxx@seu.edu.cn

badreddine.assouar@univ-lorraine.fr

**Contents**

[Supplementary Note 1. The coupling mechanism of amplitude and phase 2](#_Toc229693261)

[Supplementary Note 2. Hologram metasurface design 3](#_Toc229693262)

[Supplementary Note 3. Analysis of CAH attenuation characteristics in far-field conditions and quantitative evaluation of holographic imaging efficiency 4](#_Toc229693263)

[Supplementary Note 4. The physical mechanism of HLOA solving focusing phase distribution based on time-reversal principle 8](#_Toc229693264)

[Supplementary Note 5. High-data-density holographic reconstruction of complex topological patterns 10](#_Toc229693265)

[Supplementary Note 6. Correlation and NMSE characterization of image quality 11](#_Toc229693266)

Supplementary Note 1. The coupling mechanism of amplitude and phase

In acoustic holography, the spatial distribution characteristics of the reconstructed wavefront can be jointly described by the spatial coupling properties of the amplitude field $A\left( x \right)$ and the phase field $\phi\left( x \right)$. This coupling relationship is fundamentally determined by the wave equation of sound. The complex sound pressure can be expressed as

$P\left( x \right)=A\left( x \right)e^{i\phi\left( x \right)}$ (1)

Here, *A*(*x*) represents the amplitude distribution and *ϕ*(*x*) represents the phase distribution, both of which are functions of the spatial coordinate *x*.

To reveal the coupling mechanism between amplitude and phase, we perform a differential analysis of $P\left( x \right)$. Its first-order derivative is:

$\frac{dP\left( x \right)}{dx}=\left( \frac{dA\left( x \right)}{dx}+iA\left( x \right)\frac{d\phi\left( x \right)}{dx} \right)e^{i\phi\left( x \right)}$ (2)

The second-order derivative is:

$\frac{d^{2}P\left( x \right)}{dx^{2}}=\left( \frac{d^{2}A\left( x \right)}{dx^{2}}+2i\frac{dA\left( x \right)}{dx}\frac{d\phi\left( x \right)}{dx}+iA\left( x \right)\frac{d^{2}\phi\left( x \right)}{dx^{2}}-A\left( x \right)\left( \frac{d\phi\left( x \right)}{dx} \right)^{2} \right)e^{i\phi\left( x \right)}$ (3)

The sound pressure field satisfies the Helmholtz equation^[1]^:

$\frac{d^{2}P\left( x \right)}{dx^{2}}+k^{2}P\left( x \right)=0$ (4)

where $k=\omega/c$ is the wavenumber.

Substituting $P\left( x \right)$ and $\frac{d^{2}P\left( x \right)}{dx^{2}}$​ into the wave equation

$\left( \frac{d^{2}A\left( x \right)}{dx^{2}}+2i\frac{dA\left( x \right)}{dx}\frac{d\phi\left( x \right)}{dx}+iA\left( x \right)\frac{d^{2}\phi\left( x \right)}{dx^{2}}-A\left( x \right)\left( \frac{d\phi\left( x \right)}{dx} \right)^{2} \right)e^{i\phi\left( x \right)}+k^{2}A\left( x \right)e^{i\phi\left( x \right)}=0$ (5)

After canceling the common factor $e^{i\phi\left( x \right)}$, we obtain:

$\frac{d^{2}A\left( x \right)}{dx^{2}}+2i\frac{dA\left( x \right)}{dx}\frac{d\phi\left( x \right)}{dx}+iA\left( x \right)\frac{d^{2}\phi\left( x \right)}{dx^{2}}-A\left( x \right)\left( \frac{d\phi\left( x \right)}{dx} \right)^{2}+k^{2}A\left( x \right)=0$ (6)

Separating the real and imaginary parts yields the following coupled equations:

$\left\{ \begin{matrix} \frac{d^{2}A\left( x \right)}{dx^{2}}=A\left( x \right)\left( \left( \frac{d\phi\left( x \right)}{dx} \right)^{2}-k^{2} \right) & Real part \\ \frac{d^{2}\phi\left( x \right)}{dx^{2}}=-\frac{2}{A\left( x \right)}\frac{dA\left( x \right)}{dx}\frac{d\phi\left( x \right)}{dx} & Virtual part \end{matrix} \right.$ (7)

Real-part amplitude evolution control:

$\frac{d^{2}A\left( x \right)}{dx^{2}}-A\left( x \right)\left( \frac{d\phi\left( x \right)}{dx} \right)^{2}+k^{2}A\left( x \right)=0$ (8)

For plane wave excitation, the amplitude is constant $A\left( x \right)=A_{0}$.

$0-Ak^{2}+k^{2}A=0\Rightarrow0=0$ (9)

The phase exhibits a linear distribution.

$\phi\left( x \right)=kx+\phi_{0}\Rightarrow\frac{d\phi}{dx}=k, \frac{d^{2}\phi}{dx^{2}}=0$ (10)

Imaginary part describes phase-amplitude dynamic coupling:

$2\frac{dA\left( x \right)}{dx}\frac{d\phi\left( x \right)}{dx}+A\left( x \right)\frac{d^{2}\phi\left( x \right)}{dx^{2}}=0$ (11)

The imaginary part equation can be further rewritten in conservation form:

$\frac{d}{dx}\left( A^{2}\frac{d\phi}{dx} \right)=0$ (12)

This equation demonstrates that the sound energy density ${J\left( x \right)=A}^{2}\frac{d\phi}{dx}$ remains constant along the propagation direction^[2]^, satisfying the law of energy conservation. Under these conditions, the real part remains constant, automatically satisfying the imaginary part, thereby decoupling amplitude and phase. In non-plane wave scenarios, the imaginary part equation reflects the dynamic equilibrium between the rate of amplitude change and phase curvature, while the real part reveals the nonlinear modulation effect of phase gradients on amplitude distribution, satisfying the energy conservation theorem of Poynting^[3,4]^.

In acoustic holographic reconstruction, the accuracy of the sound field depends on the coordinated modulation of amplitude and phase. This process involves not only strong coupling effects between elements in the near-field region but also weak coupling effects arising from far-field propagation. Conservation of acoustic energy constrains the spatial distribution of coupling strength, thereby influencing imaging resolution. Extending this theory to a pixelated model, where the sound pressure at pixel $\left( i,j \right)$ is defined as $P_{ij}\left( x \right)=A_{ij}\left( x \right)e^{i\phi_{ij}\left( x \right)}$, the global coupling equation can be expressed as

$\left\{ \begin{matrix} \frac{d^{2}A_{ij}}{dx^{2}}=A_{ij}\left( \left( \frac{d\phi_{ij}}{dx} \right)^{2}-k^{2} \right), & \\ \frac{d^{2}\phi_{ij}}{dx^{2}}=-\frac{2}{A_{ij}}\frac{dA_{ij}}{dx}\frac{d\phi_{ij}}{dx}. & \end{matrix} \right.$ (13)

This coupled model provides a theoretical foundation for amplitude-phase co-modulation and can be used to guide the design and optimization of high-resolution acoustic holograms.


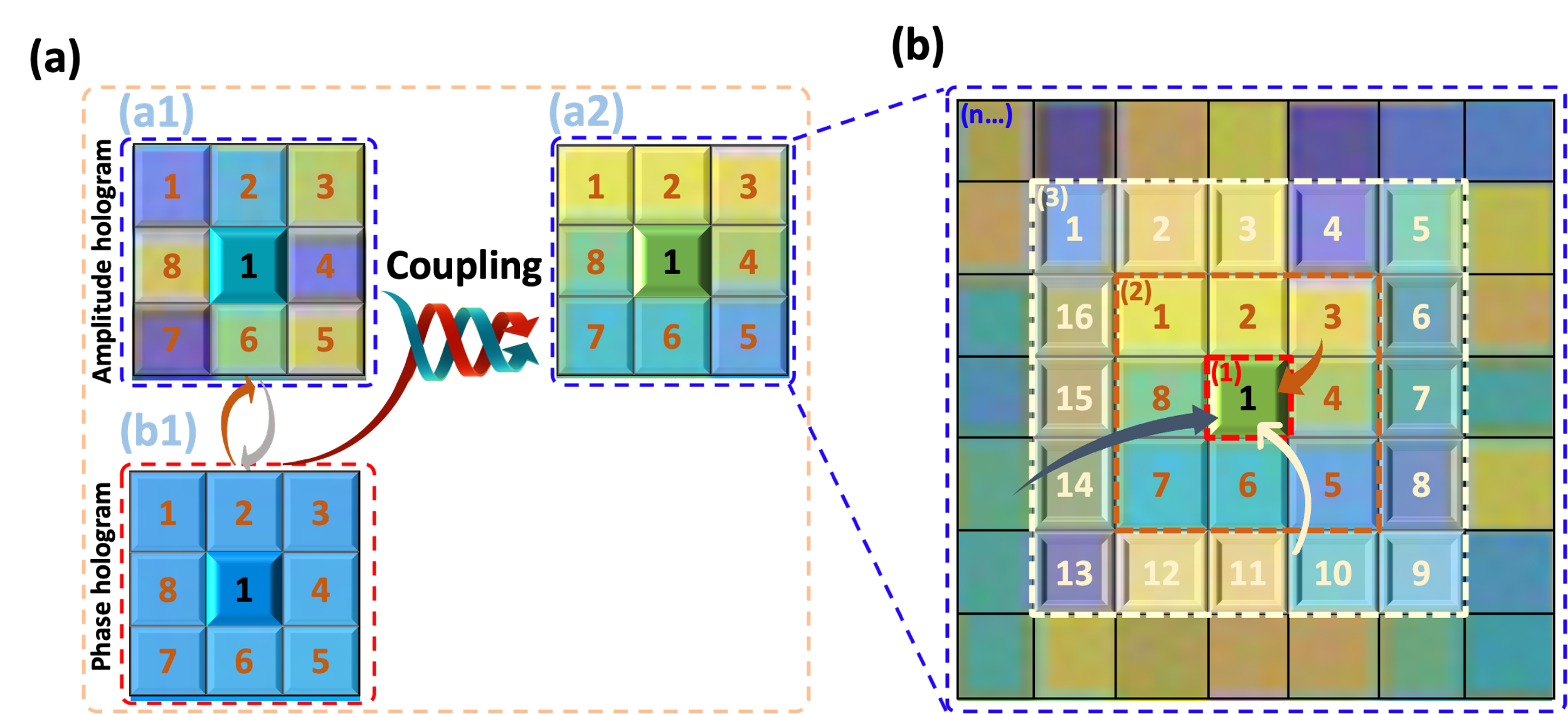


**Figure** **S1.** Local and global coupling effects. (a) a1, a2, and b1 represent local coupling effects. (b) Global pixel coupling effects on target pixels.

Supplementary Note 2. Hologram metasurface design

**Figure S2** illustrates the design process for acoustic metasurface holograms based on the amplitude-phase coupling principle, with Figure S2d specifically showing the geometric configuration of the metasurface unit cell. When plane acoustic waves incident perpendicularly, the internal structure of the metasurface can excite specific resonant modes, enabling decoupled control of both the amplitude response and phase response of the acoustic waves. This decoupling property enables each unit cell to independently modulate the amplitude and phase of the reflected sound wave. Consequently, continuous independent control over amplitude within the range [0, 1] and phase within the range [-π, π] is achieved on the holographic plane. The corresponding amplitude-phase decoupling relationships are illustrated in Figure S2e and S2f, respectively.

The acoustic metasurface unit structure comprises three parallel channels: the upper channel ($C_{1}$), middle channel ($C_{2}$), and lower channel ($C_{3}$), corresponding to heights $h_{1}$, $h_{2}$, and $h_{3}$ respectively. The widths of channels $C_{1}$ and $C_{3}$ are set to $d=\beta\cdot D$, where $\beta=0.8$, and $D=\frac{\lambda}{4}$ represents the air channel filling ratio and *λ* denotes the wavelength of sound waves in air. The total width of the unit is d. This study assumes the speed of sound in air $C_{0}=340m/s$ and an operating frequency of 1.7 kHz. The width of the middle channel C₂ is an adjustable parameter *w*, with the channel walls treated as acoustically rigid boundaries and $h_{2}$ fixed at 5 mm. By adjusting the incident channel height $h_{1}$ and the intermediate channel width *w*, independent control over the phase and amplitude of the reflected sound waves can be achieved. Figure S2e and S2f illustrate the response of the unit cell's reflected amplitude and phase to variations in parameters $h_{1}$ and *w* under decoupled conditions. The mapping relationship between $h_{1}$, *w*, and the reflected coefficient's amplitude and phase can be characterized by the following expressions based on the acoustic propagation model.

$A=\frac{d^{4}-w^{4}}{d^{4}+w^{4}}$ (14)

$\phi=-\frac{4\pi h_{1}}{\lambda}$ (15)

Taking the acoustic hologram of the letter M as an example, its corresponding amplitude distribution and the focused phase distribution obtained through reversal solution are jointly encoded within the designed acoustic metasurface. As shown in Figure S2c, this metasurface consists of periodically arranged structural units. While the external dimensions of each unit remain consistent, their internal geometric parameters, such as channel height and width, are precisely engineered based on the amplitude and phase values of corresponding pixels in the hologram. This pixel-level control design method ensures that the amplitude-phase information at every spatial point in the holographic wavefront is accurately mapped onto the metasurface structure, thereby achieving high-fidelity sound field reconstruction at the physical level.


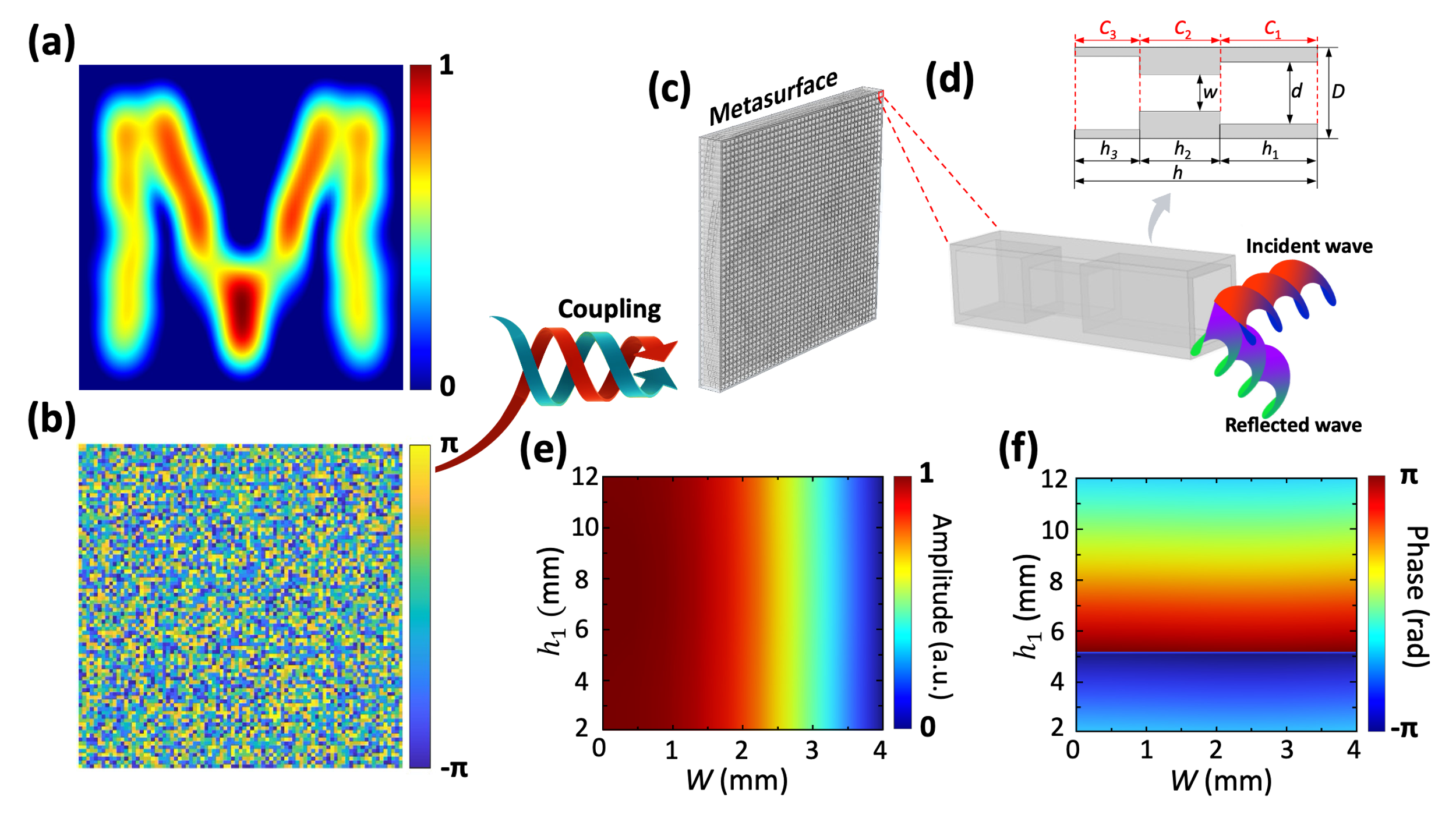


**Figure S2.** Design of amplitude-phase hologram metasurface. (a) Amplitude hologram. (b) Phase compensation distribution. (c) Metasurface of encoding amplitude hologram coupling phase compensation. (d) Unit cell structure in metasurface. (e, f) Reflection amplitude and phase response of cells to parameters $h_{1}$ and *w* under decoupling conditions.

Supplementary Note 3. Analysis of CAH attenuation characteristics in far-field conditions and quantitative evaluation of holographic imaging efficiency

**1.Analysis of Attenuation Characteristics**

The super-resolution capability of coupled holographic images exhibits three distinct phases with respect to distance:

(1) High-quality super-resolution range (0.25λ–2.5λ): Within this range, CAH can clearly resolve two subwavelength pixels into two distinct energy peaks, while maintaining a high peak-to-valley ratio, demonstrating exceptional super-resolution imaging performance.

(2) Transition zone of super-resolution performance degradation (2.75λ–3.25λ): As the imaging distance increases further, strong background noise appears in the holographic image due to the cumulative effects of acoustic wave diffraction. Although the overall contrast decreases, the two separated pixels can still be barely observed at this point.

(3) Physical Resolution Limit (>3.5λ): The effective numerical aperture (NA) characterizes the maximum range of diffraction angles that a holographic system can capture and participate in coherent superposition, directly determining the system’s ability to resolve fine features. When the imaging distance increases to 3.5λ, the geometric angle of the holographic aperture relative to the target plane significantly narrows, causing the system’s effective NA to drop sharply. This implies that the spatial frequency components of high-angle diffraction waves carrying information about subwavelength fine structures are largely lost, making effective wavefront reconstruction at the target plane impossible. Constrained by this physical mechanism, the intensity of sub-peaks with normalized heights below 0.5 undergoes severe attenuation and is completely absorbed into the main lobe, while the “trough” feature between the two peaks gradually disappears. At this point, the energy distribution of the acoustic field no longer satisfies the Rayleigh criterion or the Sparrow criterion. The two pixels completely lose their resolvability, marking the point at which the system reaches the physical limit of this super-resolution imaging method. Therefore, the effective super-resolution range of this method lies between 0.25λ and 2.5λ.

Performance Degradation Law: As shown by the quantitative curves in Figure S3, the “peak-to-valley ratio” of the CAH sound field exhibits an overall nonlinear decay trend as the propagation distance increases. The physical mechanism behind this degradation lies in the fact that, as distance increases, the strong phase gradients induced by amplitude-phase coupling can no longer tightly confine high-frequency acoustic energy over long distances within the subwavelength scale. The sound field inevitably diverges outward, leading to a decrease in image contrast and the failure of the Rayleigh criterion.


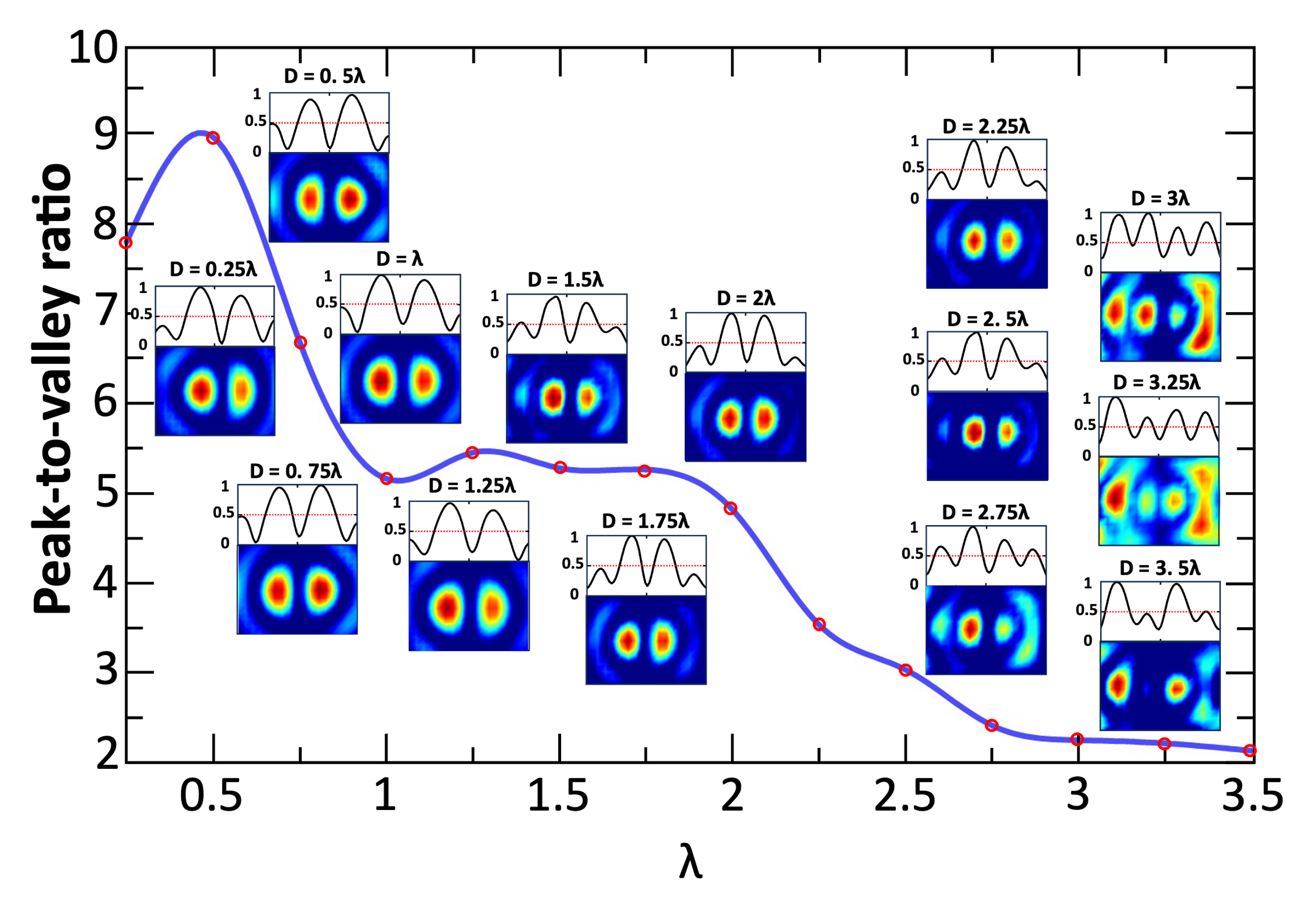


**Figure S3.** Analysis of the effective operating range and attenuation characteristics of CAH in far-field conditions.

**2. Quantitative Evaluation of Holographic Imaging Efficiency**

To quantitatively evaluate the energy transfer performance of the holographic system, this paper employs a reconstruction plane with dimensions of 44 $\times$ 44 pixels and uses an extremely small physical model—the letter “E” with a width of 1.1λ, a height of 1.5λ, and an internal gap of only 0.44λ—to perform quantitative experimental analysis and calculations of the system’s holographic diffraction and imaging efficiencies.

Based on the conservation of acoustic energy, the total acoustic energy loss during free-space propagation can be decoupled into reflection loss at the metasurface and divergence loss during spatial propagation. Therefore, the total holographic imaging efficiency ($\eta_{total}$) is defined as the product of the amplitude modulation efficiency ($\eta_{amp}$) and the spatial focusing efficiency ($\eta_{focus}$).

$\eta_{total}=\eta_{amp}\times\eta_{focus}$ (16)

The amplitude modulation efficiency ($\eta_{amp}$) is defined as the ratio of the total acoustic energy actually reflected by the metamaterial surface to the total acoustic energy incident under uniform conditions. In a discretized acoustic field model, the acoustic energy is proportional to the square of the sound pressure amplitude.

$\eta_{amp}= \frac{\sum\left| A_{holo}(i,j) \right|^{2}}{N_{holo}}$ (17)

Here, $A_{holo}(i,j)$ represents the normalized reflection amplitude of each discrete element on the metamaterial surface, and $N_{holo}$ denotes the total number of pixels on the holographic surface. Since the amplitude-phase coupling modulation mechanism requires the introduction of local amplitude attenuation to generate the phase gradients necessary for breaking the diffraction limit, the reflection efficiency calculated under this physical modulation mechanism is 35.03%.

Defining the spatial focusing efficiency ($\eta_{focus}$) as the ratio of the acoustic energy effectively concentrated within the target letter “E” region to the total acoustic energy distributed across the entire imaging plane. In a discrete acoustic field model, the sound intensity is proportional to the square of the absolute value of the complex sound pressure $I=\frac{|p|^{2}}{2\rho_{0}c_{0}}$. Therefore, using the ratio of the square of the sound pressure to evaluate efficiency can be expressed as

$\eta_{focus}= \frac{\sum_{(x,y)\in\Omega_{target}} \left| p_{j}(x,y) \right|^{2}}{\sum_{(x,y)\in\Omega_{total}} \left| p_{j}(x,y) \right|^{2}}$ (18)

Here, $p_{j}(x,y)$ represents the complex sound pressure distribution on the reconstructed image plane, $\Omega_{target}$ denotes the integration region for the letter “E” target, and $\Omega_{total}$ denotes the integration region for the entire imaging plane. Based on these values, the spatial focusing efficiency is calculated to be 45.06%.

Based on the above decoupling analysis, the total holographic imaging efficiency of the system is calculated to be 15.79%. Analysis of the results shows that the amplitude-phase coupled modulation system achieves 45% subwavelength spatial resolution by sacrificing 65% of the reflected energy. This loss in overall energy efficiency is an inherent physical cost of the amplitude-phase coupled system in achieving subwavelength spatial resolution.

We can observe that the internal sound intensity distribution of the letter “E” in the reconstructed sound field exhibits a non-uniform pattern, with a brighter center and darker edges. This phenomenon is essentially determined by the combined effects of the coherent superposition envelope at the wavelength scale and the inherent physical trade-offs of super-resolution imaging. On the one hand, since the overall physical dimensions of the microscopic target region are close to the wavelength scale, the reconstructed sound field is inevitably constrained macroscopically by the overall diffraction energy envelope. According to the Huygens–Fresnel principle, diffracted waves from the holographic plane exhibit the smallest path difference and the strongest coherent superposition when they reach the central region of the target. In contrast, in the peripheral regions, increased energy dissipation due to wide-angle diffraction naturally causes the sound pressure amplitude to attenuate. On the other hand, to resolve minute gaps within a finite physical aperture, the amplitude-phase coupling modulation mechanism must concentrate the limited high-spatial-frequency components to construct steep phase gradients at structural boundaries and induce strong destructive interference. The cost of this regulation, which prioritizes achieving ultimate spatial resolution, inevitably compromises the uniformity of the internal sound energy distribution, ultimately resulting in an acoustic envelope characterized by constructive interference at the center and diffraction-induced attenuation at the edges.

To further validate the aforementioned physical mechanism, this paper conducted systematic comparative validation experiments. First, the spatial bandwidth product of the image plane was expanded from 44 $\times$ 44 pixels to 60 $\times$ 60 pixels; while keeping the absolute physical size of the letter “E” constant, the non-uniformity in the reconstructed sound field energy distribution persisted. Subsequently, while keeping the spatial bandwidth product at 44 $\times$ 44 pixels, the physical size of the target letter “E” was doubled, expanding the width of its internal gap to 0.88λ, as shown in Figure S4. The reconstruction results at this stage showed that, as the target size gradually moved away from the subwavelength limit, the acoustic energy distribution within the letter exhibited significant homogenization. The above comparative results fully demonstrate that this non-uniformity in the intensity distribution is not a system artifact caused by the discretization of the computational matrix, but rather an intrinsic physical response independent of the system’s spatial bandwidth product and determined entirely by the absolute physical scale of the target structure relative to the wavelength.


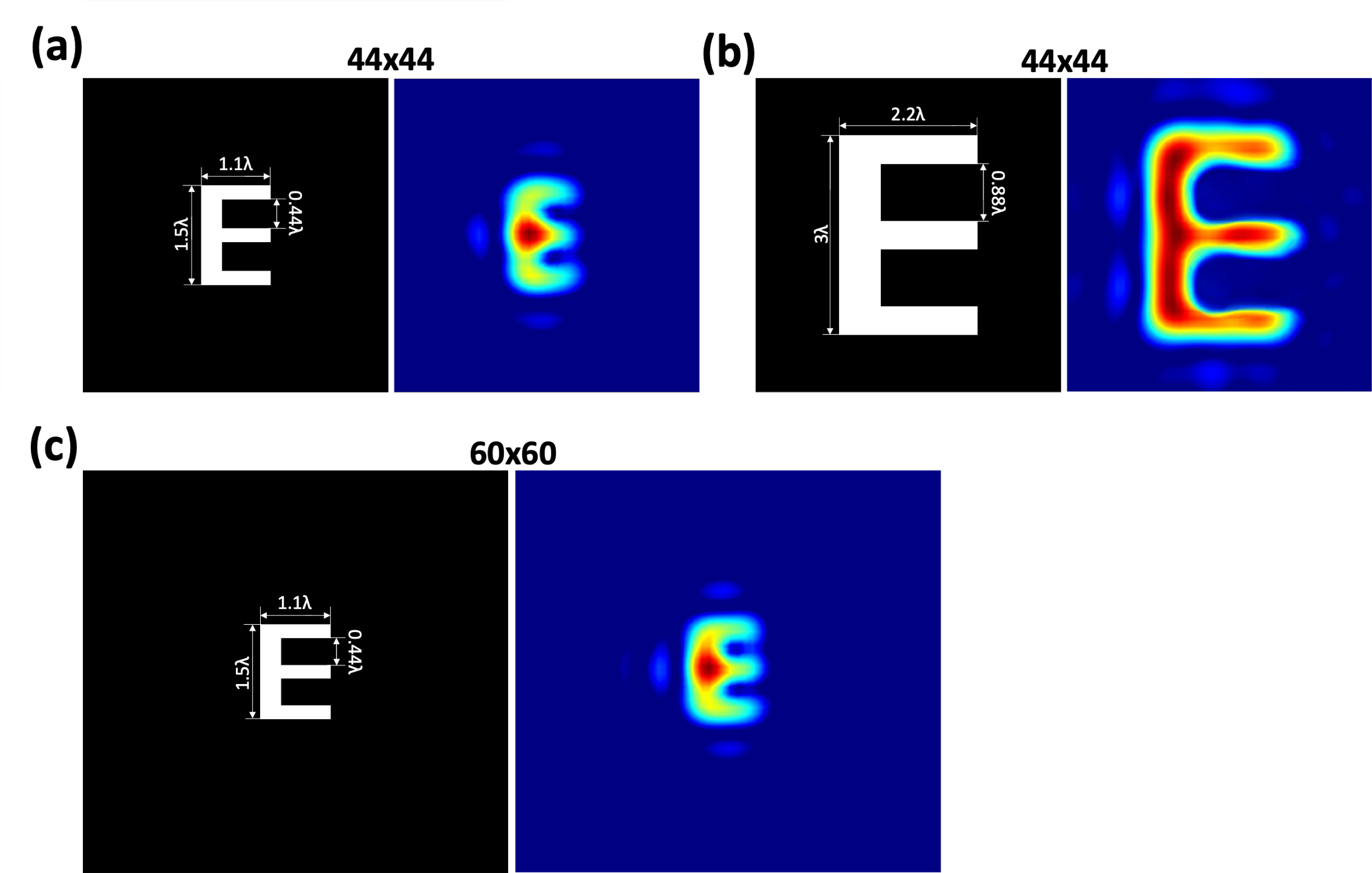


**Figure S4.** A comparative analysis of the effects of target physical scale and system spatial bandwidth product on the uniformity of reconstructed sound field distribution. (a) The reconstructed sound field when the image plane spatial bandwidth product is 44 $\times$ 44 pixels and the target is at the subwavelength scale (characteristic gap 0.44λ); (b) Reconstructed sound field when the target’s absolute physical scale is doubled (characteristic gap 0.88λ) while maintaining the image plane spatial bandwidth product at 44 $\times$ 44 pixels; (c) Reconstructed sound field when the image plane spatial bandwidth product is expanded to 60 $\times$ 60 pixels while maintaining the subwavelength target scale of the letter E.

Supplementary Note 4. The physical mechanism of HLOA solving focusing phase distribution based on time-reversal principle

In acoustic holographic imaging, the target image can be discretized into a pixel array at the subwavelength scale. Based on the principle of time-reversal, wavefront components generated by each pixel on the image plane can undergo coherent superposition on the holographic plane^[5]^, thereby reconstructing the sound field distribution characterized by the complex sound pressure $P_{j}=A_{j}exp({i\phi}_{j})$. Here, $A_{j}$ and $\phi_{j}$ represent the amplitude and phase at the *j*-th position on the holographic plane, respectively. This reconstruction process can be described by the following expression:

$p_{j}=\sum_{l=1}^{N} \frac{A_{0l}}{r_{l}}\exp\left[ i\left( k_{0}r_{l}+\phi_{0l} \right) \right]\equiv A_{j}\exp\left( i\phi_{j} \right)$ (16)

Here, *N* denotes the total number of pixels in the image, $A_{0l}$and $\phi_{0l}$ represent the initial amplitude and phase at the *l*-th pixel in the image plane, respectively, and *k*_0_ is the wave number. $r_{l}$ indicates the Euclidean distance between the *j-*th position on the holographic surface and the *l*-th pixel in the image plane, given by the following equation:

$r_{l}=\sqrt{\left( x_{j}-x_{l} \right)^{2}+\left( y_{j}-y_{l} \right)^{2}+\left( z_{j}-z_{l} \right)^{2}}$ (17)

Based on the symmetry of acoustic time inversion, the aforementioned reconstruction process can be reverse-projected to directly generate a predefined acoustic hologram. The sound pressure at any spatial point (*x, y, z*) in the hologram can be expressed as:

$P\left( x,y,z \right)=\sum_{j=1}^{n} \frac{A_{j}}{r_{j}}\exp\left[ -i\left( k_{0}r_{j}-\phi_{j} \right) \right]$ (18)

where n is the total number of pixels in the hologram, and $r_{j}$ is the distance between the spatial point $\left( x,y,z \right)$ and the holographic pixel point $\left( x_{j},y_{j},z_{j} \right)$, defined as:

$r_{j}=\sqrt{\left( x-x_{j} \right)^{2}+\left( y-y_{j} \right)^{2}+\left( z-z_{j} \right)^{2}}$ (19)

This expression is essentially a mathematical formulation of the Huygens-Fresnel principle under discrete sound source conditions, embodying the physical picture of spherical wave superposition during wavefront reconstruction.

To enhance the resolution of acoustic holographic imaging under low pixel density conditions, this study employs the Horned Lizard Optimization Algorithm (HLOA) based on the principle of acoustic energy conservation to reconstruct the focal plane phase distribution $\varphi_{0l}$ on the target imaging plane. HLOA integrates five biomimetic behavioral strategies, iteratively solving for the phase distribution that maximizes both hologram imaging resolution and reconstruction accuracy.

Strategy 1: Crypsis behavior. Simulating the camouflage characteristics of horned lizards, the position update formula for the *i*-th agent (i.e., candidate phase distribution) is

$\vec{x}_{i}\left( t+1 \right)=\vec{x}_{best}\left( t \right)+\left( \partial-\frac{\partial\cdot t}{{Max}_{iter}} \right)\cdot\left[ c_{1}\left( sin\left( \vec{x}_{c}\left( t \right) \right)-cos\left( \vec{x}_{r_{2}}\left( t \right) \right) \right)-\left( -1 \right)^{\sigma}c_{2}\left( cos\left( \vec{x}_{r_{3}}\left( t \right) \right)-sin\left( \vec{x}_{r_{4}}\left( t \right) \right) \right) \right]$ (20)

where $\vec{x}_{best}\left( t \right)$  represents the current optimal phase distribution, $c_{1}$​ and $c_{2}$​ denote normalization adjustment parameters, and $r_{1}$​, $r_{2}$​, $r_{3}$​, $r_{4}$​​ are random indices. This strategy balances global exploration and local exploitation through the oscillatory behavior of trigonometric functions.

Strategy 2: Skin darkening or lightening. To replace the suboptimal phase distribution, an adaptive skin tone strategy is introduced.

$\vec{x}_{worst}\left( t \right)=\vec{x}_{best}\left( t \right)+\frac{1}{2}Light_{1}sin\left( \vec{x}_{r_{1}}\left( t \right)-\vec{x}_{r_{2}}\left( t \right) \right)-\left( -1 \right)^{\sigma}\frac{1}{2}Light_{2}sin\left( \vec{x}_{r_{3}}\left( t \right)-\vec{x}_{r_{4}}\left( t \right) \right)$ (21)

$\vec{x}_{worst}\left( t \right)={\overset{\to}{x}}_{best}\left( t \right)+\frac{1}{2}Dark_{1}sin\left( {\overset{\to}{x}}_{r_{1}}\left( t \right)-{\overset{\to}{x}}_{r_{2}}\left( t \right) \right)-\left( -1 \right)^{\sigma}\frac{1}{2}Dark_{2}sin\left( {\overset{\to}{x}}_{r_{3}}\left( t \right)-{\overset{\to}{x}}_{r_{4}}\left( t \right) \right)$ (22)

where $Light_{1}$,$Light_{2}\in[0, 0.4046661]$ and $Dark_{1}$,$Dark_{2}\in[0.5440510, 1]$. This operation enhances population diversity by perturbing the worst-performing agents.

Strategy 3: Blood-squirting. Simulates the parabolic trajectory of a horned lizard's blood spray to achieve strong global exploration capabilities:

$\vec{x}_{i}\left( t+1 \right)=\left[ v_{o}cos\left( \alpha\frac{t}{Max_{i}ter} \right)+\varepsilon\right]\vec{x}_{best}\left( t \right)+\left[ v_{o}sin\left( \alpha-\frac{\alpha t}{Max_{i}ter} \right)-c+\varepsilon\right]\vec{x}_{i}\left( t \right)$ (23)

where $v_{0}=1m/s$ , $\alpha=\frac{\pi}{2}$, $g=0.009807km/s^{2}$ This strategy effectively escapes local optima through dynamic trajectory updates.

Strategy 4: Move-to-escape. Balances local fine-grained search with global exploration.

$\vec{x}_{i}\left( t+1 \right)=\vec{x}_{best}\left( t \right)+walk\left( \frac{1}{2}-\varepsilon\right)\vec{x}_{i}\left( t \right)$ (24)

where $walk\in[-1, 1]$ and *ϵ* follows a Cauchy distribution, enabling the search to favor the vicinity of the current optimal solution.

Strategy 5: α-melanophore stimulating hormone (α-MSH) rate. Dynamically adjust the individual renewal probability based on fitness values.

$melanophore\left( i \right)=\frac{Fitness_{max}-Fitness\left( i \right)}{Fitness_{max}-Fitness_{min}}$ (25)

where $Fitness_{\max}$ and $Fitness_{\min}$ are the best and worst fitness values in the current T-generation, respectively, while $Fitness\left( i \right)$ is the current fitness value of the *i-th* search agent. When the *α*-MSH rate is defined as less than 0.3, replacing the search agent can be represented by the following equation, where $Fitness\left( i \right)$quantifies amplitude imaging quality. When $melanophore\left( i \right)<0.3$, update the agent.

$\vec{x}_{i}\left( t \right)=\vec{x}_{best}\left( t \right)+\frac{1}{2}\left[ \vec{x}_{r_{1}}\left( t \right)-\left( -1 \right)^{\sigma}\vec{x}_{r_{2}}\left( t \right) \right]$ (26)

This mechanism prioritizes replacing phase distribution schemes with lower fitness. For the loss function design, this method achieves super-resolution imaging of holograms under low information density by coupling the phase distribution of the amplitude hologram with the focus through HLOA-based acoustic energy conservation reversal. The average difference between the amplitude distribution *A_a_*_​_(*x, y*) of the planar target and the actual reconstructed amplitude *A_d_*(*x, y*) serves as the loss function, driving the phase reversal process.

$Loss= \frac{1}{N}\sum_{x,y} [A_{d}\left( x,y \right)-A_{a}\left( x,y \right)]$ (27)

To further validate the optimization performance of HLOA, this study introduced the classical Particle Swarm Optimization (PSO) algorithm as a benchmark under the same parameter settings and compared the resulting hologram images after reversal coupling and optimization of the focusing phase distribution, as shown in Figure S5. In terms of sound field reconstruction quality and solution accuracy, a comparison of the converged holographic sound field distributions (inset) reveals that the holograms obtained after solving the coupled focusing phase using HLOA can more accurately reconstruct the target pattern, demonstrating significantly improved spatial resolution and more complete image contours. Regarding convergence dynamics and global search performance, the PSO algorithm’s curve flattens after approximately 200 iterations, exhibiting typical premature convergence characteristics and making it prone to getting trapped in local optima within the fitness landscape, with the reconstruction error ultimately stagnating at 24.3; In contrast, HLOA, leveraging its superior global search mechanism and ability to escape local optima, maintains a stepwise decrease in fitness values throughout the entire iteration cycle, effectively breaking through the optimization plateau. Quantitative results indicate that HLOA achieves a deeper level of global optimization, further reducing the final convergence loss to 22.4, representing a significant reduction of approximately 7.8% compared to PSO. The above results fully demonstrate that, when addressing such complex acoustic inverse problems, HLOA significantly outperforms traditional mainstream swarm intelligence optimization algorithms in terms of solution accuracy, global optimization depth, and algorithmic robustness.


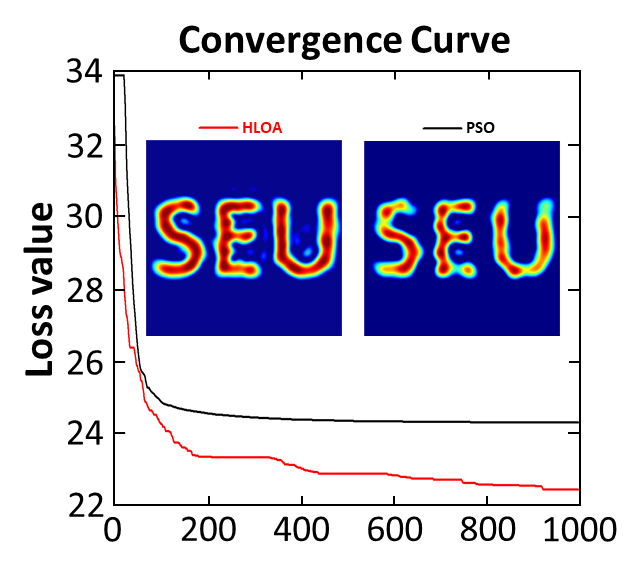


**Figure S5.** Convergence curves for HLOA and PSO.

Supplementary Note 5. High-data-density holographic reconstruction of complex topological patterns

In the main body of this study, we not only used simple geometric letters and deep subwavelength double-point arrays as imaging targets to precisely extract the full width at half maximum (FWHM) of the acoustic field distribution, thereby quantitatively verifying the physical mechanism of subwavelength super-resolution, but also demonstrated the system’s ability to process complex intersecting topologies at the experimental level through localized Crani patterns. To further explore the theoretical processing limits of the proposed amplitude-phase coupling method for images with extremely high data density, this section introduces complex targets—such as butterflies and clovers—with broad-band spatial features and multi-scale continuous curve contours for comparative analysis.

Given that reconstructing such complex patterns with dense textures requires a large scalar bandwidth-bandwidth product. We employed a 230 $\times$ 230-pixel bandwidth imaging plane and conducted physical experiments at an operating frequency of 17 kHz. However, the required physical aperture of the metasurface was too large, exceeding the capabilities of conventional laboratory-scale high-precision fabrication and full-field scanning measurements. Therefore, in this section, we validate the complex patterns through rigorous full-wave numerical simulations to assess the applicability of the amplitude-phase coupling modulation mechanism to complex topological information.

Figure S6 compares the reconstruction results of complex patterns using conventional amplitude holograms (AH) and the amplitude-phase coupled holograms (CAH) proposed in this paper. Under the same spatial bandwidth product constraint of 230 $\times$ 230 pixels, due to the lack of effective compensation for the focusing phase gradient, the conventional AH (Fig. S6b) exhibits significant diffraction aliasing when processing fine structures with high spatial frequencies, particularly in the butterfly’s antennae, the internal patterns on its wings, and the continuous curved edges of the cloverleaf. This results in severe degradation of image details.

In contrast, CAH (Fig. S6c) enhances the utilization efficiency of the finite number of physical degrees of freedom N2 by introducing local amplitude-phase coupling and energy conservation mechanisms, thereby achieving effective control over high-frequency spatial components. The results demonstrate that CAH effectively focuses extremely high-density complex topological textures while maintaining the smoothness and structural integrity of complex curved contours. These numerical validation results indicate that, provided the highest spatial frequency of the target sound field does not exceed the maximum phase gradient limit that the CAH array can provide, this mechanism possesses the physical potential to reconstruct complex continuous sound fields with high data density.

**
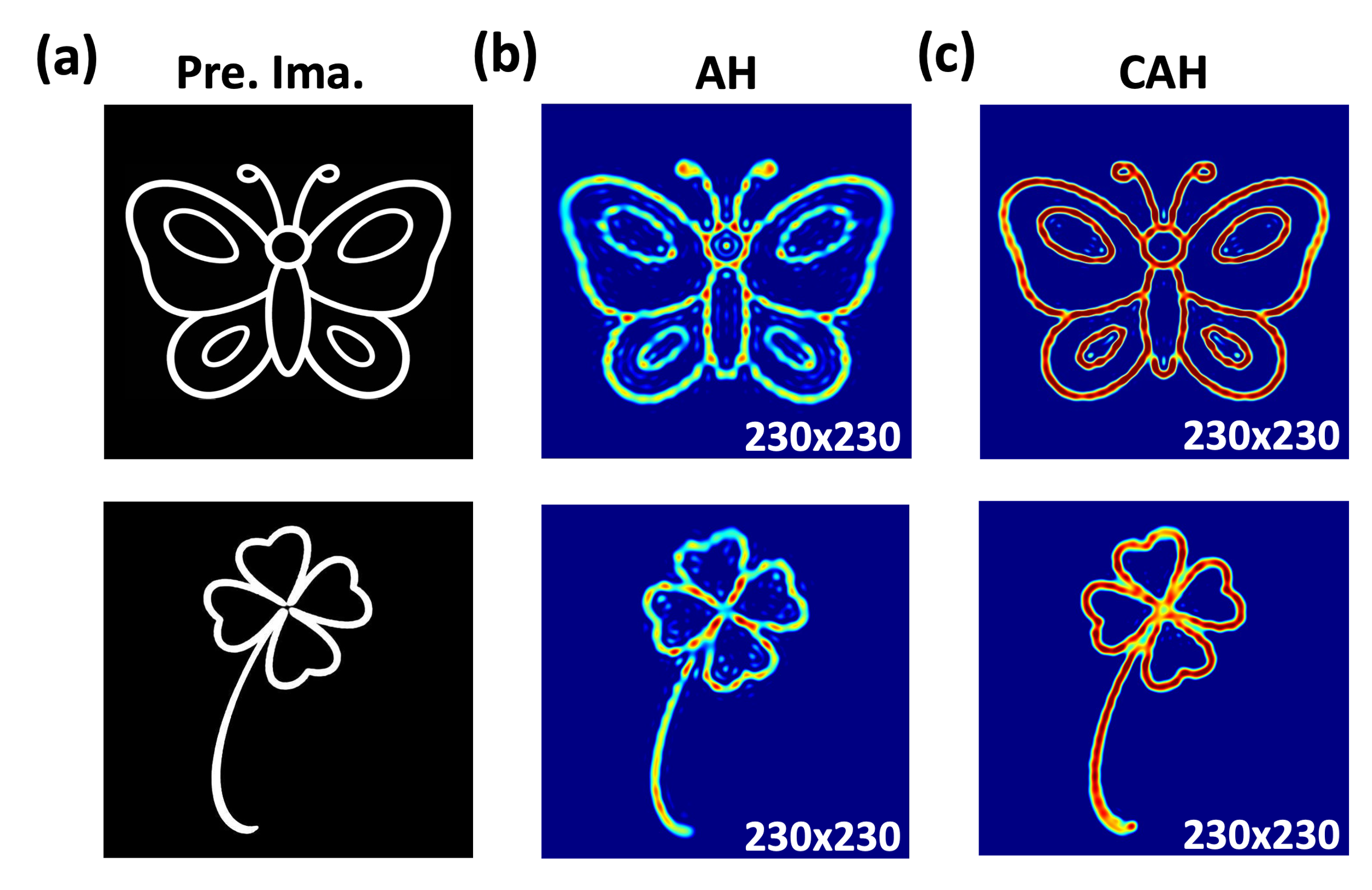
**

**Figure S6.** Comparison of high-data-density holographic reconstructions of complex images. (a) Pre-designed butterfly and four-leaf clover target images. (b) Reconstruction results based on conventional amplitude holograms (AH). (c) Reconstruction results based on amplitude-phase-coupled holograms (CAH).

Supplementary Note 6. Correlation and NMSE characterization of image quality

The correlation for evaluating the similarity between two images is calculated by

$\text{Correlation}=\frac{\sum_{j} \sum_{i} \left( P_{i,j}-P \right)\left( H_{i,j}-H \right)}{\sqrt{\left( \left( \sum_{j} \sum_{i} \left( P_{i,j}-P \right)^{2} \right)\left( \sum_{j} \sum_{i} \left( H_{i,j}-H \right)^{2} \right) \right)}}$ (28)

In the context where *P* represents the target image and *H* denotes the holographic image, with $P$ and $H$ indicating the mean values of the respective images.

The Normalized Mean Square Error (NMSE) is expressed as

$NMSE=\frac{\left\| P-H \right\|_{2}^{2}}{\left\| P \right\|_{2}^{2}}$ (29)

Here *P* represents the theoretical hologram and *H* denotes the fabricated hologram, with $\left\| \cdot\right\|_{2}$ denoting the Euclidean norm. The NMSE for super-resolution and high-data-density holographic imaging can be illustrated by **Figure S7a** and **S7b**.


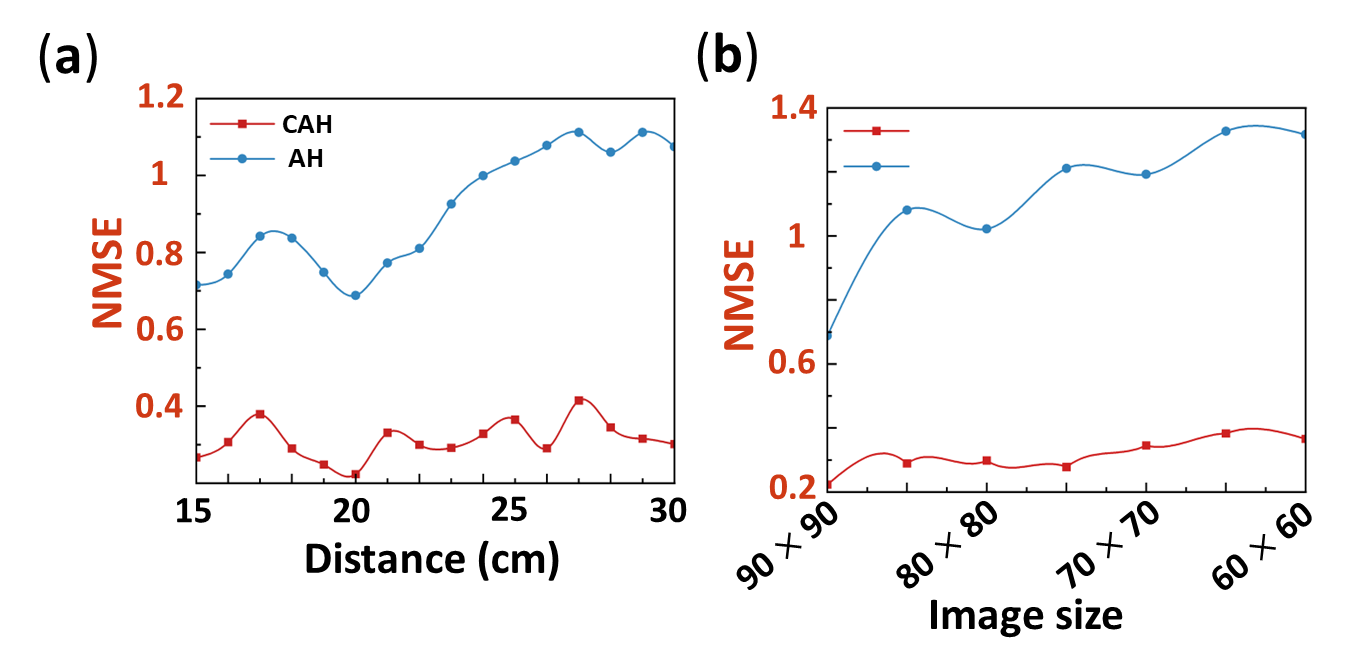


**Figure S7.** NMSE for super-resolution and high-data-density holographic imaging. (a) NMSE for super-resolution holographic imaging. (b) NMSE for high-data-density holographic imaging.

**References**

[1] S. Wang, M. V. De Hoop, J. Xia, *J. Comput. Phys.* **2010**, *229*, 8445.

[2] P. J. Westervelt, The Journal of the Acoustical Society of America **1963**, *35*, 535.

[3] V. Domínguez-Rocha, C. Zagoya, M. Martínez-Mares, *Am. J. Phys.* **2008**, *76*, 621.

[4] P. Kinsler, A. Favaro, M. W. McCall, *Eur. J. Phys.* **2009**, *30*, 983–993.

[5] Y. Zhu, J. Hu, X. Fan, J. Yang, B. Liang, X. Zhu, J. Cheng, *Nat. Commun.* **2018***, 9*, 1632.

[6] H. Peraza-Vázquez, A. Peña-Delgado, M. Merino-Treviño, A. B. Morales-Cepeda, N. Sinha, *Artif. Intell. Rev.* **2024**, *57*, 59.
